# Role of Alkaline-Earth Metal-Catalyst: A Theoretical Study of Pyridines Hydroboration

**DOI:** 10.3389/fchem.2019.00149

**Published:** 2019-03-26

**Authors:** Yuanyuan Li, Meijun Wu, Haohua Chen, Dongdong Xu, Lingbo Qu, Jing Zhang, Ruopeng Bai, Yu Lan

**Affiliations:** ^1^Department of Biological and Chemical Engineering, Chongqing University of Education, Chongqing, China; ^2^Cooperative Innovation Center of Lipid Resources and Children's Daily Chemicals, Chongqing University of Education, Chongqing, China; ^3^College of Chemistry and Molecular Engineering, ZhengZhou University, ZhengZhou, China; ^4^School of Chemistry and Chemical Engineering, Chongqing University, Chongqing, China; ^5^Department of Chemistry and Chemical Engineering, Jining University, Jining, China

**Keywords:** alkaline-earth-metals catalyst, theoretical study, hydroboration, dihydropyridine, metal hydride complex

## Abstract

Density functional theory (DFT) calculations have been performed to investigate the mechanism of alkaline-earth-metal-catalyzed hydroboration of pyridines with borane. In this reaction, the active catalytic species is considered to be an alkaline earth metal hydride complex when the corresponding alkaline earth metal is used as the catalyst. The theoretical results reveal that initiation of the catalytic cycle is hydride transfer to generate a magnesium hydride complex when β-diimine alkylmagnesium is used as a pre-catalyst. The magnesium hydride complex can undergo coordination of the pyridine reactant followed by hydride transfer to form a dearomatized magnesium pyridine intermediate. Coordination of borane and hydride transfer from borohydride to magnesium then give the hydroboration product and regenerate the active magnesium hydride catalyst. The rate-determining step of the catalytic cycle is hydride transfer to pyridine with a free energy barrier of 29.7 kcal/mol. Other alkaline earth metal complexes, including calcium and strontium complexes, were also considered. The DFT calculations show that the corresponding activation free energies for the rate-determining step of this reaction with calcium and strontium catalysts are much lower than with the magnesium catalyst. Therefore, calcium and strontium complexes can be used as the catalyst for the reaction, which could allow mild reaction conditions.

## Introduction

As an important derivative of pyridine, dihydropyridine is an important raw material to synthesize natural products. For instance, dihydropyridine is extensively used to synthesize nicotinamide adenine dinucleotide, antihypertensive drugs, and anti-inflammatory agents (Karrer et al., 1937; Mauzerall and Westheimer, 1955; Bossert et al., 1981; Schramm et al., 1983; Berg et al., 2002). In addition, because dihydropyridine can provide two hydrogen atoms, it is often used as a mild and efficient reducing agent with high selectivity in catalytic hydrogenation (Adolfsson, 2005; Connon, 2007). Dihydropyridine can be prepared by dearomatization of pyridine and its derivatives. However, application of this reaction is limited by its strict reaction conditions and unstable dearomatized intermediates (Hantzsch, 1881; Stout and Meyers, 1982; Seyferth, 2009; Li Y. Y. et al., 2018). Synthesis of these compounds by homogeneous catalysis has seldom been reported (Harrod et al., 2001; Oshima et al., 2012a,b). In 1998, Harrod's research group (Hao et al., 1998) realized pyridine hydrosilylation and obtained the 1,2-dihydropyridine product with a titanocene derivative as the catalyst. In 2011, Nikonov's group (Gutsulyak et al., 2011) realized hydrosilylation of pyridine with ruthenium as the catalyst at room temperature and obtained a mixture of 1,2- and 1,4-borohydropyridine. However, application of these reactions in synthetic chemistry is greatly limited by the expensive transition metal catalyst (Yaroshevsky, 2006; Dobereiner and Crabtree, 2010; Osakada, 2011; Huang and Xia, 2015; Li et al., 2015; Qi et al., 2016, 2018; Yang et al., 2016; Yu et al., 2016; Xing et al., 2017; Liu et al., 2018; Luo et al., 2018; Zhu et al., 2018).

Alkaline-earth (Ae) metals, which are located in group IIA of the periodic table of elements, have attracted wide attention because of their low cost, availability, and abundant reserves in nature (Green et al., 2007). Ae metals, such as magnesium, calcium, strontium, and barium, are commonly used as homogeneous catalysts. Because the d^0^ valence electron configuration at the center of the divalent cation (Ae^2+^) in the Ae metal catalyst shows partial “lanthanide” characteristics, a similar catalytic cycle can be constructed in homogeneous catalysis (Li and Marks, 1996; Westerhausen, 2008; Krieck et al., 2009; Hill et al., 2016; Ma et al., 2016; Rochat et al., 2016; Rossin and Peruzzini, 2016; Xu et al., 2017a).

In the past few decades, many research groups have prepared series of organic Ae metal compounds, which have wide application prospects owing to their low cost and toxicity (Arrowsmith et al., 2011; Liu et al., 2012; Intemann et al., 2014; Schwamm et al., 2014; Hill et al., 2016; Rossin and Peruzzini, 2016). One of the most important applications is the hydrogen transfer reaction, in which Ae metal hydrides are usually used to transfer hydrogen atoms and they show unique catalytic activity (Dunne et al., 2011; Harder et al., 2011; Praneeth et al., 2012; Intemann et al., 2013; Liptrot et al., 2014; Anker et al., 2015; Weetman et al., 2016). The high catalytic activity of Ae metal hydrides can be ascribed to two factors. First, the hydrogen atoms in the Ae metal hydride have high electron density, which means that the hydrogen atoms can easily dissociate in the form of free hydride anions that exhibit Brønsted basicity. Second, Ae metal hydrides also exhibit Lewis acidity owing to the two formal positive charges carried by the metal ions (Rokob et al., 2013; Anker et al., 2014; Stepha, 2015; Hill et al., 2016). For example, the magnesium catalyst can catalyze the hydroboration reaction of borohydride compounds (pinacol—borane (HBpin), 9-borabicyclo [3.3.1]nonane (9-BBN), etc.) with various organic compounds (esters, ketones, amines, pyridines, imines, nitriles, and amides) to construct new C (hetero)–B bonds (Barrett et al., 2007; Hill et al., 2010; Arrowsmith et al., 2012; Butera et al., 2014; Schwamm et al., 2014; Lampland et al., 2015; Liptrot et al., 2015; Liu et al., 2017; Zhao et al., 2017; Jiang et al., 2018).

**Scheme 1:**
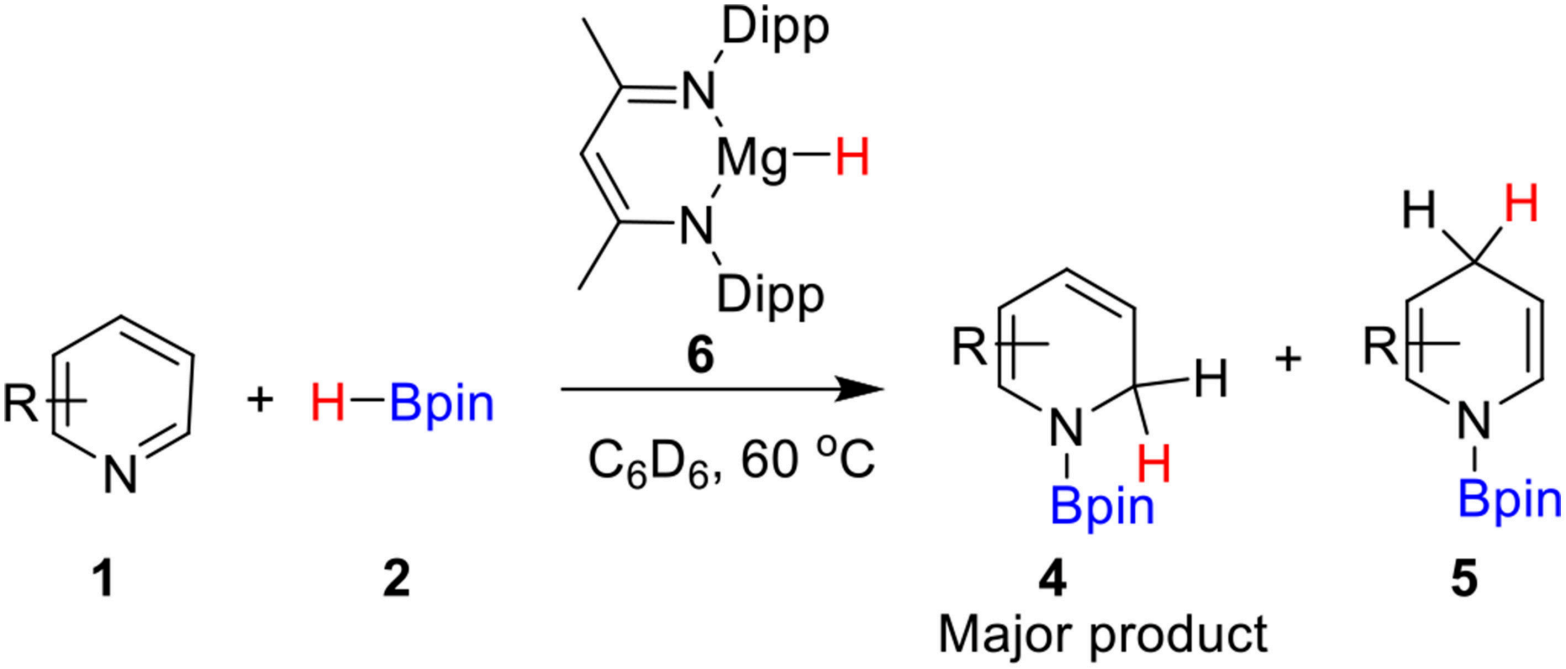
Magnesium-catalyzed hydroboration of pyridines with borane.

**Scheme 2:**
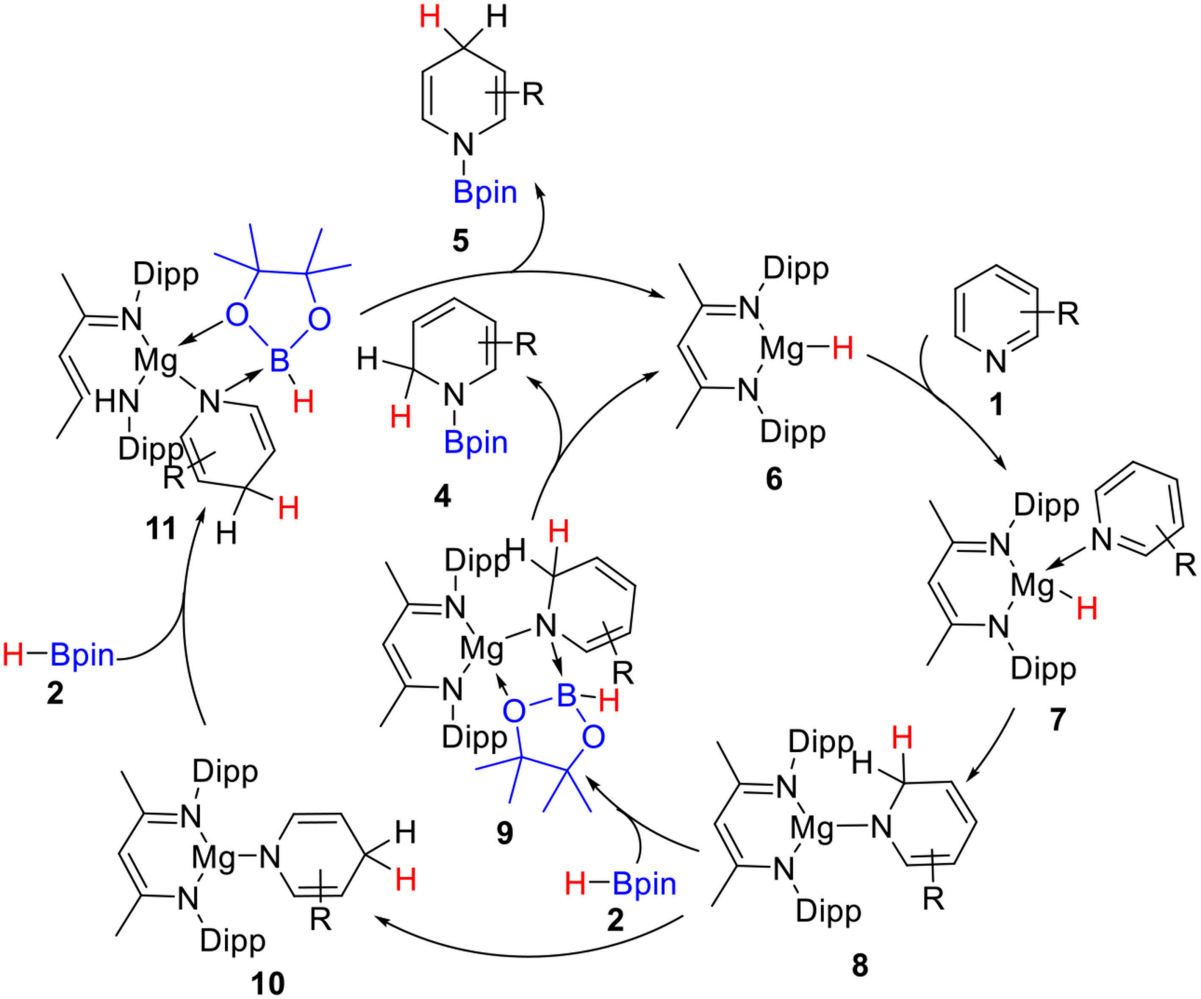
Plausible mechanism for magnesium-catalyzed hydroboration of pyridines with borane.

Hill and co-workers (Arrowsmith et al., 2011) first reported the magnesium-catalyzed hydroboration of Pyridines in 2011. In 2014, Harder's group (Intemann et al., 2014) reported hydroboration of pyridine catalyzed by the Ae metal magnesium and obtained the 1,2-selective addition compound **4** as the major product (Scheme 1). Harder et al. suggested that magnesium hydride species **6** acts as a catalyst in the catalytic cycle. Based on the experimental observations and our previous theoretical studies of Ae catalysis, there are two possible reaction pathways. Raw material pyridine **1** coordinates with magnesium in active catalytic species **6** to give intermediate **7** (Scheme 2). The C = N double bond in pyridine then inserts into the Mg–H bond to give dearomatized magnesium amino intermediate **8**, which could coordinate with pinacolborane **2** to give nitrogen–boron compound **9**. Finally, magnesium hydride species **6** is regenerated by hydride transfer with the release of 1,2-borohydropyridine derivative **4**. Alternatively, intermediate **8** could be isomerized by 1,3-hydrogen migration to give intermediate **10**, which then coordinates to pinacolborane **2** to give nitrogen–boron complex **11**. After the corresponding hydride transfer, 1,4-borohydropyridine product **5** is obtained and active magnesium hydride species **6** is regenerated.

Here, we performed a theoretical mechanistic study of Ae catalysis and determined the trend of the reactivates of Ae-catalyzed hydrogenation reactions. The pyridine hydroboration reaction catalyzed by Ae metals reported by Harder's group was used as the template reaction for the theoretical calculations. The calculations were performed based on density functional theory (DFT) and the reaction mechanism is discussed to better understand the reaction process and provide theoretical guidance for subsequent organic reactions catalyzed by Ae metals.

## Computational Methods

All of the DFT calculations were performed with the Gaussian 09 software package (Frisch et al., 2013). The B3-LYP (Lee et al., 1988; Becke, 1993; Stephens et al., 1994; Adamo and Barone, 1999) functional with the standard 6-31G(d) (Hehre et al., 1972; Francl et al., 1982) basis set (SDD Dolg et al., 1987 basis set for strontium atoms) was used for the geometry optimizations. Harmonic vibrational frequency calculations were performed for all of the stationary points to determine whether they are local minima or transition structures and to derive the thermochemical corrections for the enthalpies and free energies. The M11 (Peverati and Truhlar, 2011) functional with the 6-311+G(d) basis set (SDD for strontium atoms) was used to calculate the single-point energies, because it is expected that this strategy will provide more accuracy with regard to the energetic information (Peverati and Truhlar, 2012; Zhao et al., 2012; Yu and Lan, 2013; Cui et al., 2019). The solvent effect of benzene was considered by single-point calculations of the gas-phase stationary points with the solvation model based on the density (SMD), which is a continuous model (Cances et al., 1997; Marenich et al., 2009). The reported free energies are the M11-calculated Gibbs free energies in benzene solvent based on the B3-LYP-calculated geometries with thermodynamic corrections. The geometric configurations of the key reaction intermediates and transition states were generated with CYLview software (Legault, 2009). The total energies for all of the calculated structures are listed in Supplementary Material.

## Results and Discussion

### Mechanism of Pyridine Hydroboration Catalyzed by Magnesium-Hydrogen Species

**Figure 1:**
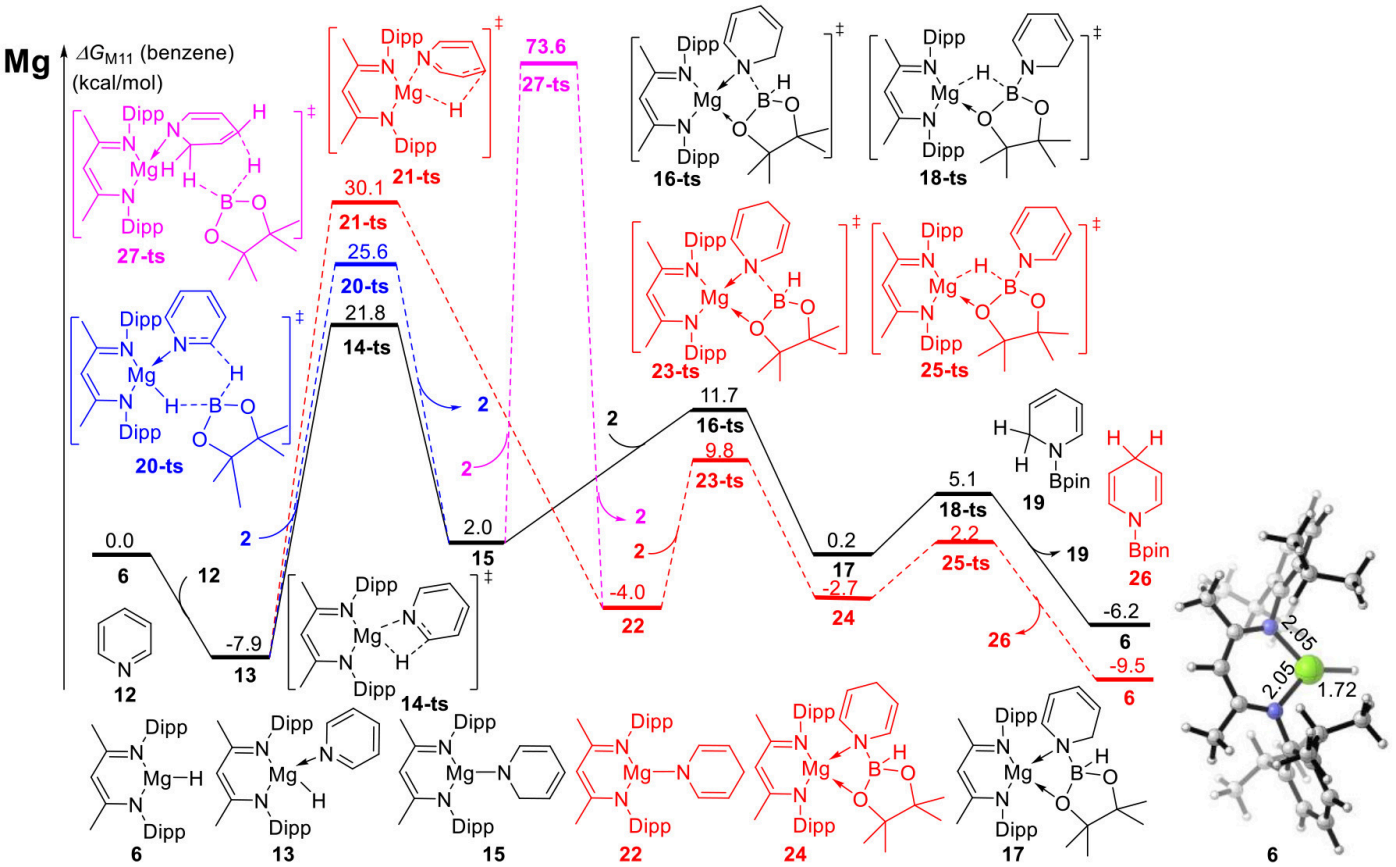
Free energy profile for magnesium-catalyzed hydroboration of pyridines and borane. The relative free energies (ΔG) in benzene are given in kcal/mol.

**Figure 2:**
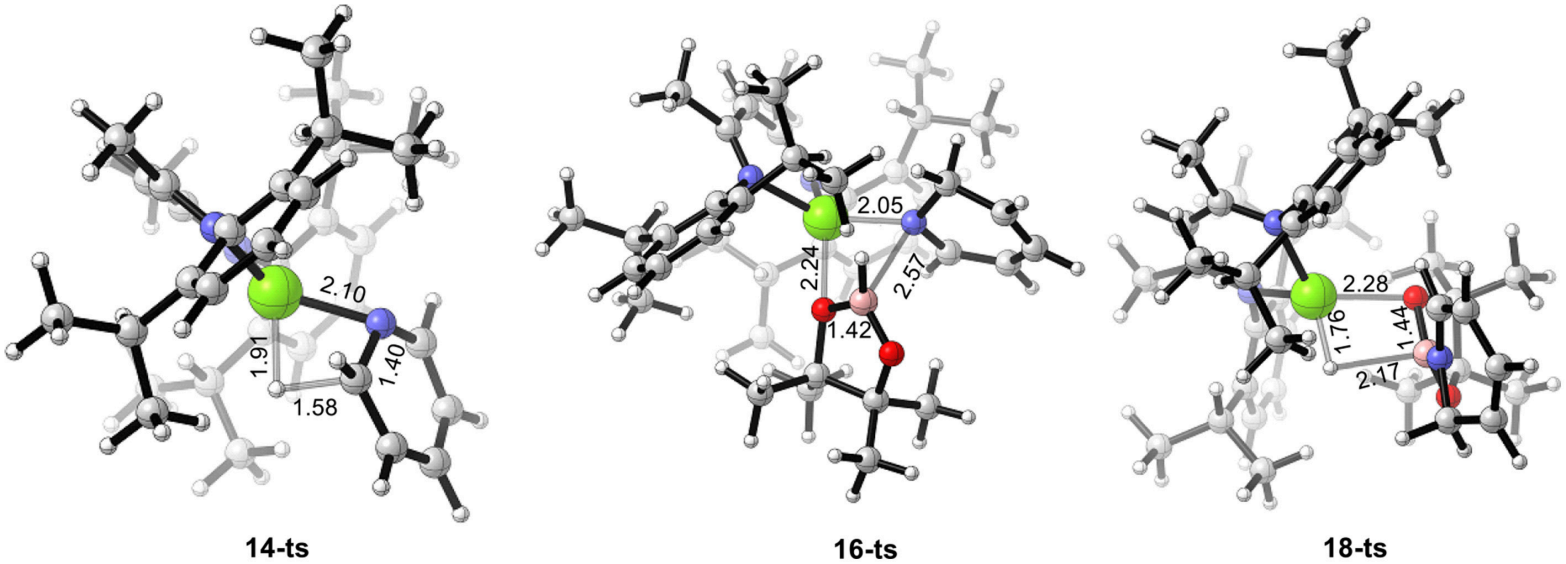
Geometry of transition states **14-ts**, **16-ts**, and **18-ts**. The bond lengths are given in ångstroms.

DFT calculations were performed for hydroboration of pyridines catalyzed by magnesium (Scheme 2). We selected β-diimine magnesium hydride **6** as the relative zero point of the Gibbs free energy (Figure 1). Pyridine **12** coordinates with active magnesium hydride species **6** to form intermediate **13**. This process is exergonic by 7.9 kcal/mol, indicating that the magnesium hydride species can be stabilized as the Lewis acid by the corresponding Lewis base. After pyridine is activated by the magnesium hydride species, the C = N double bond in pyridine **12** inserts into the Mg–H bond via transition state **14-ts**. the energy barrier of insertion step is 29.7 kcal/mol, indicating that this step is the rate-determining step of the catalytic cycle. The geometric structure of transition state **14-ts** is shown in Figure 2. The length of the C–H bond to be formed is 1.58 Å. The length of the Mg–H bond to be ruptured is 1.91 Å. The length of the C–N bond in pyridine involved in the reaction is 1.40 Å. The above data of the geometrical structure show that formation of the C–H bond and rupture of the Mg–H bond simultaneously occur in this reaction. Intermediate **15** is obtained when C = N double insertion occurs. Because the aromaticity of pyridine in intermediate **15** is destroyed, its relative energy is 9.9 kcal/mol higher than that of intermediate **13**. In addition, we also considered another route to obtain intermediate **15**. With pinacolborane **2** acts as a bridge, the hydrogen atom migrates from magnesium to boron via hexatomic ring transition state **20-ts** and then transfers from boron to the ortho carbon of nitrogen to give intermediate **15**. The activation energy of this process is 33.5 kcal/mol, which indicates that it is a possible alternative pathway to generate intermediate **15**. In intermediate **15**, because the Mg–N covalent bond is formed and there is a lone pair of electrons on the nitrogen atom, it can coordinate with pinacolborane **2** to give boron–nitrogen Lewis acid–base complex **17** via low-energy transition state **16-ts**. Interestingly, in intermediate **17**, there are also two lone pairs of electrons on each oxygen atom in pinacolborane **2**, so a coordination bond can form between oxygen and magnesium, increasing the stability of intermediate **17**. The pinacolborane **2** moiety of intermediate **17** is activated by the amino group, so hydride can migrate from boron to magnesium. Theoretical calculations show that this process proceeds via transition state **18-ts** with an activation free energy of only 5.3 kcal/mol. After this step, 1,2-borohydropyridine product **19** is released and active catalytic species **6** is regenerated. The complete catalytic cycle is only exothermic by 6.2 kcal/mol. This indicates a weak thermodynamic driving force, which can be attributed to the aromaticity of pyridine being destroyed. Nevertheless, a stable N–B covalent bond is generated to drive the reaction.

### Mechanism of Pyridine Hydroboration Catalyzed by Other Ae Metals

**Figure 3:**
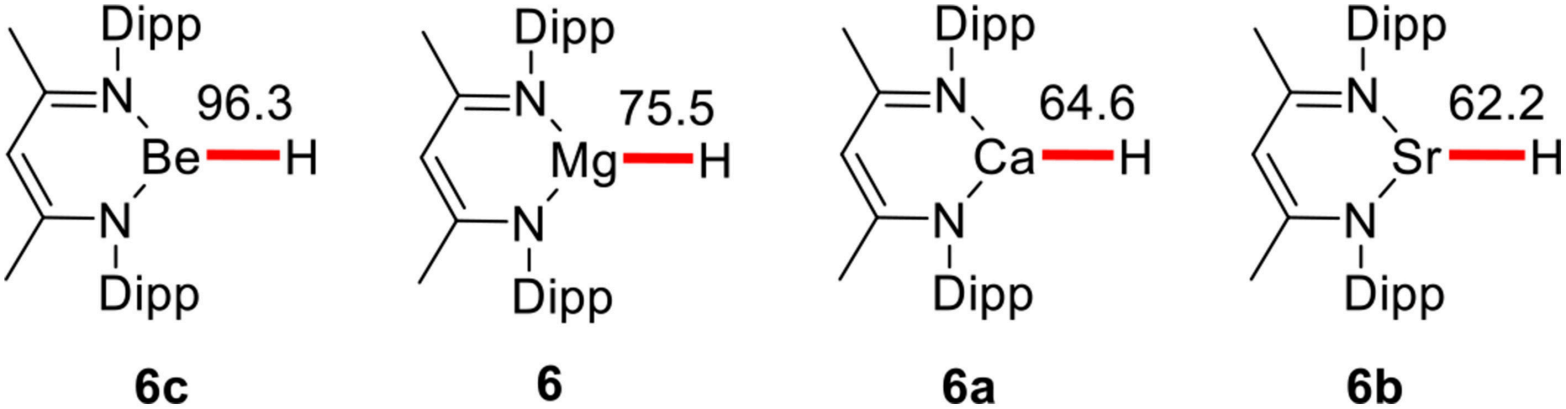
The value of BDEs for corresponding Ae–H bonds given in kcal/mol. Free energies (ΔG) in benzene are given in kcal/mol.

Harder's group only determined the catalytic activities of magnesium species. Followed these results, Harder and co-workers (Intemann et al., 2015) reported the calcium catalyzed hydrosilylation of pyridine, demonstrating that a beta-deketiminate calcium hydride is capable of faster selective 1,2-dearomatisation in 2015. According to the basic rule of the periodic law of elements and our previous theoretical studies (Gao et al., 2016; Qi et al., 2017; Xu et al., 2017a,b; Li, Y. et al., 2018) elements of the same main group often have similar chemical properties (Cotton et al., 1988). After investigating the mechanism of pyridine hydroboration catalyzed by the magnesium catalyst, we performed M11 calculations to predict the catalytic activities of other Ae metals in the group. In the pyridine hydroboration reaction, the rate-determining step is considered to be pyridine C = N bond insertion with Mg–H bond cleavage. Therefore, a weak metal–H covalent bond is favorable for this reaction. The calculated bond dissociation energies (BDEs) of Ae–H bonds are shown in Figure 3. The BDEs of Be–H, Mg–H, Ca–H, and Sr–H are 96.3, 75.5, 64.6, and 62.2 kcal/mol, respectively. Therefore, beryllium, which is in the same group as magnesium, has a small atomic radius, large steric hindrance, low metallic property, and strong Be–H bond. We believe that hydroboration cannot be catalyzed by beryllium hydride species. Furthermore, calcium and strontium could have higher catalytic activity than magnesium because of the weak metal–H covalent bonds owing to their larger atomic radii and lower electronegativities.

**Figure 4:**
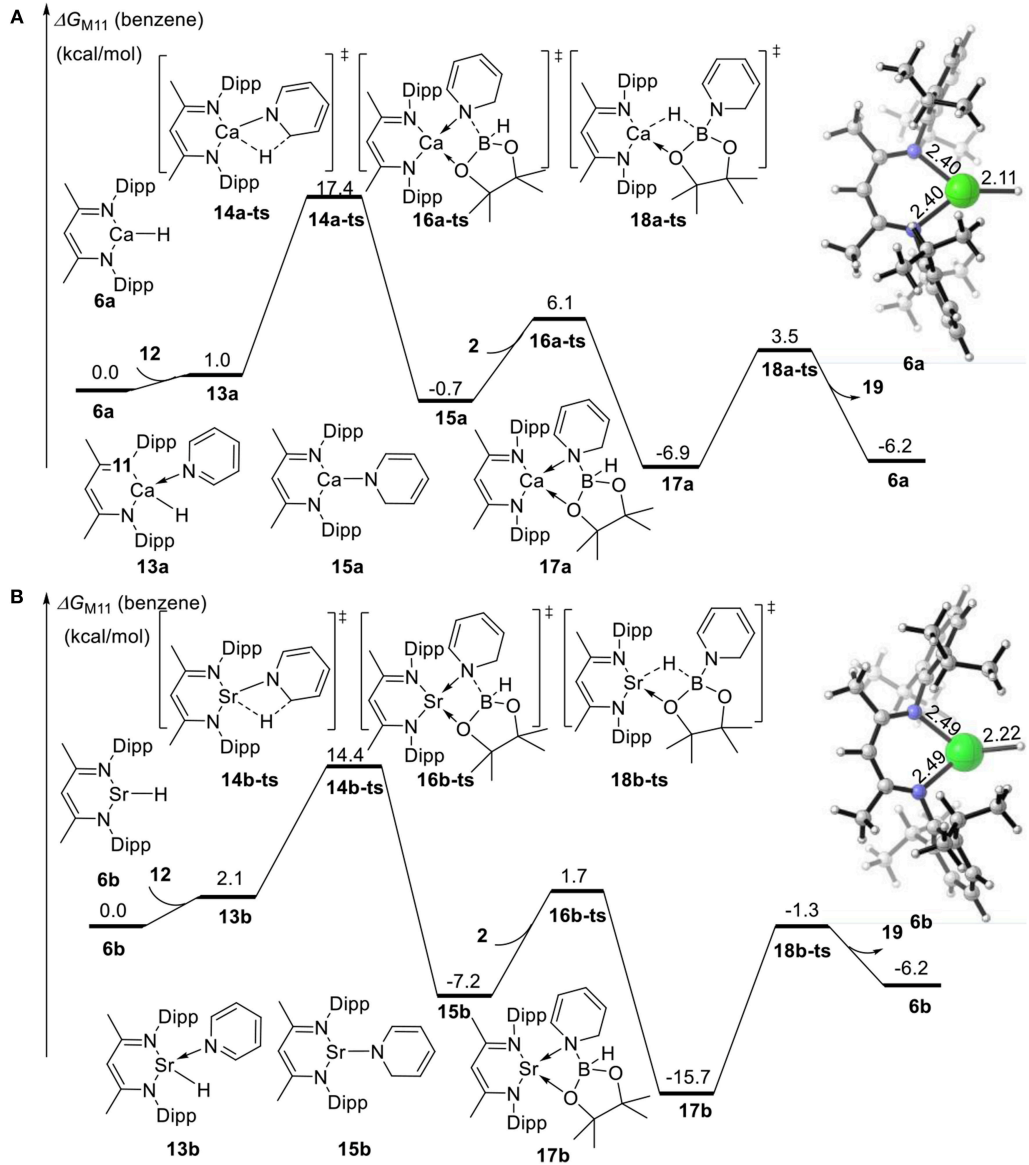
Free energy profile for calcium-catalyzed **(A)** or strontium-catalyzed **(B)** hydroboration of pyridines and borane. The relative free energies (ΔG) in benzene are given in kcal/mol.

**Figure 5:**
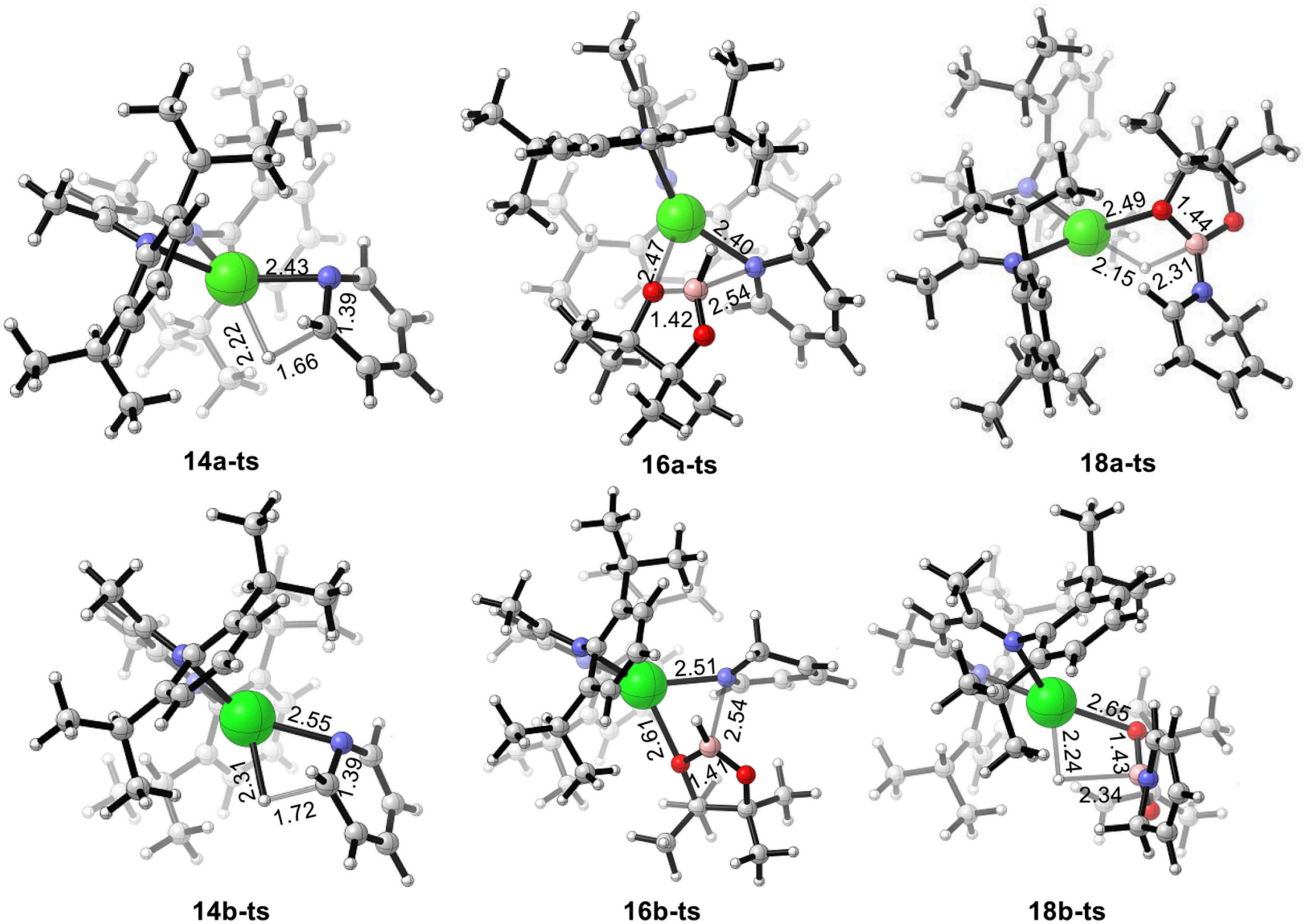
Geometry of transition states **14a-ts**, **16a-ts**, **18a-ts**, **14b-ts**, **16b-ts**, and **18b-ts**. The bond lengths are given in ångstroms.

We also calculated the potential energy surfaces of the pyridine hydroboration reaction catalyzed by calcium and strontium hydride (Figure 4). In Figure 4A, active calcium hydride catalyst **6a** is selected as the relative zero of the Gibbs free energy. First, calcium hydride species **6a** coordinates with pyridine **12** to give intermediate **13a** with a relative Gibbs free energy of 1.0 kcal/mol. The pyridine C = N bond then inserts into the Ca–H covalent bond via transition state **14a-ts**. The energy barrier of this process is 17.4 kcal/mol, indicating that this step is the rate-determining step of the catalytic cycle. Compared with the corresponding reaction catalyzed by magnesium, the activation energy required for the rate-determining step decreases by 12.3 kcal/mol. Thus, we can conclude that the activity of the calcium catalyst is higher than that of the magnesium catalyst. Intermediate **15a** coordinates with pinacolborane **2** via the corresponding low-energy transition state **16a-ts** to give boron–nitrogen Lewis acid–base complex **17a**. Finally, 1,2-borohydropyridine product **19** is released by hydride transfer with regeneration of active catalytic species **6a**. The geometric structures of **14a-ts**, **16a-ts**, and **18a-ts** are shown in Figure 5. The corresponding Ae metal transition states have similar structures.

Strontium hydride species **6b** can also coordinate with pyridine **12** to give intermediate **13b**, which is endergonic by 2.1 kcal/mol (Figure 4B). Intermediate **13b** is unstable and prone to react. The activation energy required for C = N bond insertion into the Sr–H bond via transition state **14b-ts** is 14.4 kcal/mol. However, intermediate **17b** generated by coordination to pinacolborane **2** is very stable, and its free energy is 9.5 kcal/mol lower than that of the 1,2-borohydropyridine product **19** and regenerated active catalyst **6b**. Therefore, the complete activation free energy for strontium catalysis of this reaction is 23.9 kcal/mol, which is 5.8 kcal/mol lower than that for magnesium catalysis, but 5.8 kcal/mol higher than that for calcium catalysis. Therefore, our theoretical calculations predict that the catalytic activities of the Ae metal complexes are calcium > strontium > magnesium.

## Conclusion

The mechanism of pyridine hydroboration catalyzed by a series of Ae metals has been systematically investigated by DFT calculations. When a magnesium complex is used as the catalyst, the magnesium hydride intermediate is the active catalytic species for this reaction. According to the theoretical calculations, the reaction mechanism of this reaction is as follows. Pyridine first coordinates with the magnesium atom in the active catalyst, followed by the pyridine C = N double bond inserting into the Mg–H bond, resulting in dearomatization. The magnesium amino intermediate coordinates to pinacolborane and the magnesium hydride intermediate is regenerated by hydride transfer to release the product. The catalytic activities of various Ae metals in the same group were predicted by DFT calculations. The trend of the Ae–H BDEs is Mg–H > Ca–H > Sr–H. The theoretical calculations indicate that when a calcium catalyst is used, there is a much lower activation free energy for C = N double bond insertion into the Ca–H bond, which is the rate-determining step of the catalytic cycle. However, because the boron–nitrogen Lewis acid–base complex with the strontium catalyst is very stable, the apparent activation free energy for the strontium catalyst is higher than that for the calcium catalyst, but lower than that for the magnesium catalyst. Therefore, we believe that milder reaction conditions could be used if a calcium or strontium complex is used as the catalyst.

## Author Contributions

The work was completed by cooperation of all authors. YyL, RB, and YL were responsible for the study of concept and design of the project. MW and HC searched the intermediates and transition states, JZ and DX analyzed the data and drew energy profiles. YyL, LQ, and YL drafted and revised the manuscript.

### Conflict of Interest Statement

The authors declare that the research was conducted in the absence of any commercial or financial relationships that could be construed as a potential conflict of interest.
