# Supplementary material for: Role of Alkaline-Earth Metal-Catalyst: A Theoretical Study of Pyridines Hydroboration
Source: Front Chem. 2019 Mar 26;7:149. doi: 10.3389/fchem.2019.00149 (PMC6443636; doi:10.3389/fchem.2019.00149)
Supplement: Supplementary file 1 [file Data_Sheet_1.doc]

Supplementary Material

**Role of alkaline-earth metal-catalyst: a theoretical study of pyridines hydroboration** *Supporting Information*

Yuanyuan Li^1,2,3^, Meijun Wu^1^, Haohua Chen^4^, Dongdong Xu^4^, Lingbo Qu^3^, Jing Zhang^5^, Ruopeng Bai^4*^and Yu Lan^3,4*^

*^1^ Department of Biological and Chemical Engineering, Chongqing University of Education, Chongqing 400067, People`s Republic of China.*

*^2^ Cooperative Innovation Center of Lipid Resources and Children’s Daily Chemicals, Chongqing University of Education, Chongqing 400067, People`s Republic of China.*

*^3^ College of Chemistry and Molecular Engineering, ZhengZhou University, ZhengZhou, 450006, People`s Republic of China.*

*^4^ School of Chemistry and Chemical Engineering, Chongqing University, Chongqing, 401331, People`s Republic of China.*

*^5^ Department of Chemistry and Chemical Engineering, JiNing University, JiNing, 272001, People`s Republic of China.*

**Table of contents**

1. Completes reference for Gaussian 09.………………………………………………………………………Page S2

2. Computational Methods.…………………………………………………………………………………………Page S2

3. The hydroboration mechanism of pyridine catalyzed by magnesium-hydrogen species.…Page S2

4. The hydroboration mechanism of pyridine catalyzed by other alkaline-earth metals………Page S3

5. The value of BDE…………………………………………………………………………………………………..PageS4

6. Absolute calculation energies, enthalpies, and free energies.………………………………………Page S4

7. B3LYP geometries for all the optimized compounds and transition states. ……………………Page S6

**1. Complete reference for Gaussian 09**

Gaussian 09, Revision A.2, Frisch, M. J.; Trucks, G. W.; Schlegel, H. B.; Scuseria, G. E.; Robb, M. A.; Cheeseman, J. R.; Scalmani, G.; Barone, V.; Mennucci, B.; Petersson, G. A.; Nakatsuji, H.; Caricato, M.; Li, X.; Hratchian, H. P.; Izmaylov, A. F.; Bloino, J.; Zheng, G.; Sonnenberg, J. L.; Hada, M.; Ehara, M.; Toyota, K.; Fukuda, R.; Hasegawa, J.; Ishida, M.; Nakajima, T.; Honda, Y.; Kitao, O.; Nakai, H.; Vreven, T.; Montgomery, Jr., J. A.; Peralta, J. E.; Ogliaro, F.; Bearpark, M.; Heyd, J. J.; Brothers, E.; Kudin, K. N.; Staroverov, V. N.; Kobayashi, R.; Normand, J.; Raghavachari, K.; Rendell, A.; Burant, J. C.; Iyengar, S. S.; Tomasi, J.; Cossi, M.; Rega, N.; Millam, N. J.; Klene, M.; Knox, J. E.; Cross, J. B.; Bakken, V.; Adamo, C.; Jaramillo, J.; Gomperts, R.; Stratmann, R. E.; Yazyev, O.; Austin, A. J.; Cammi, R.; Pomelli, C.; Ochterski, J. W.; Martin, R. L.; Morokuma, K.; Zakrzewski, V. G.; Voth, G. A.; Salvador, P.; Dannenberg, J. J.; Dapprich, S.; Daniels, A. D.; Farkas, Ö.; Foresman, J. B.; Ortiz, J. V.; Cioslowski, J.; Fox, D. J. Gaussian, Inc., Wallingford CT, **2013**.

**2. Computational Methods.**

All the DFT calculations were carried out with the GAUSSIAN 09 series of programs. Density functional theory B3LYP^1^ with a standard 6-31G(d) basis set was used for geometry optimizations. Harmonic frequency calculations were performed for all stationary points to confirm them as a local minima or transition structures and to derive the thermochemical corrections for the enthalpies and free energies. M11^2^ functional with basis set 6-311+G(d,p) was employed to calculate the solvation single point energies to give more accurate energy information (SDD for strontium atoms). The solvent effects were considered by single point calculations on the gas-phase stationary points with a SMD continuum solvation model^3^. The energies given in this paper are M11 calculated Gibbs free energies.

**References:**

1. (a) Lee, C.; Yang, W.; Parr, R. G. *Phys. Rev. B* **1988**, *37*, 785; (b) Becke, A. D. *J. Chem. Phys.* **1993**, *98*, 5648.

2. (a) Peverati, R.; Truhlar, D. G. *Phys. Chem. Chem. Phys.* **2012**, *14*, 16187; (b) Peverati, R.; Truhlar, D. G. *J. Phys. Chem. Lett.* **2012**, *3*, 117; (c) Zhao, Y.; Ng, H. T.; Peverati, R.; Truhlar, D. G. *J. Chem. Theory Comput.* **2012**, *8*, 2824; (d) Yu, Z.; Lan, Y. *J. Org. Chem.* **2013**, *78*, 11501;

3. Marenich, A. V.; Cramer, C. J.; Truhlar, D. G. *J. Phys. Chem. B* **2009**, *113*, 6378.

**3. The hydroboration mechanism of pyridine catalyzed by magnesium-hydrogen species.**

Mechanistic study using a larger basis set might take a lot of time, however there’s no great difference and using a smaller basis set could improve work efficiency. Considering the reviewer’s comments, we re-calculated an advantage path yet with a larger basis set 6-311+G(d, p). And the comparison of free energy profile (Figure S1) shows that there’s indeed no obvious difference between the computational results obtained by basis set 6-311+G(d, p) and 6-31G(d). Additionally, the related reference, which supported that the previous mixed basis set could afford a good performance, was also cited in the revised manuscript. The corresponding calculation results are shown below:

**Figure S1**. Potential energy surface for magnesium catalysed hydroboration of pyridines and borane The energies are in kcal/mol and represent the relative free energies, which were calculated using the M11 method in benzene solvent.

**4. The hydroboration mechanism of pyridine catalyzed by other alkaline-earth metals.**

**Figure S2**. Potential energy surface for calcium (a) or strontium (b) catalysed hydroboration of pyridines and borane. The energies are in kcal/mol and represent the relative free energies, which were calculated using the M11 method in benzene solvent.

**5. The value of BDE**

**Figure S3.** The value of BDEs. The BDEs are given in kcal/mol.

**6. Absolute calculation energies, enthalpies, and free energies.**

**Table S1**. Absolute calculation energies, enthalpies, and free energies

| Geometry | E_(elec-B3LYP)_^1^ | G_(corr-B3LYP)_^2^ | H_(corr-B3LYP)_^3^ | E_(solv,M11)_^4^ | IF^5^ |
| --- | --- | --- | --- | --- | --- |
| **2** | -411.863861171 | 0.158372 | 0.202007 | -411.759508162 |  |
| **6** | -1440.02739216 | 0.574688 | 0.682256 | -1439.59004705 |  |
| **12** | -248.27966994 | 0.06169 | 0.094301 | -248.200271579 |  |
| **13** | -1688.32909551 | 0.66017 | 0.77928 | -1687.82670366 |  |
| **14-ts** | -1688.28723191 | 0.658862 | 0.777762 | -1687.77805794 | -827.58 |
| **15** | -1688.32003561 | 0.664321 | 0.783291 | -1687.81517864 |  |
| **16-ts** | -2100.1723926 | 0.849795 | 0.986403 | -2099.58624851 | -65.01 |
| **17** | -2100.19588167 | 0.853894 | 0.988916 | -2099.60871613 |  |
| **18-ts** | -2100.1791594 | 0.847121 | 0.984364 | -2099.59406732 | -168.17 |
| **19** | -660.164679008 | 0.243644 | 0.300575 | -659.993312308 |  |
| **20-ts** | -2100.160235 | 0.842677 | 0.982333 | -2099.557036 | -628.77 |
| **21-ts** | -1688.27202872 | 0.662149 | 0.777172 | -1687.76816537 | -807.51 |
| **22** | -1688.32596029 | 0.661814 | 0.783452 | -1687.8221394 |  |
| **23-ts** | -2100.1807173 | 0.851941 | 0.987218 | -2099.59147807 | -145.89 |
| **24** | -2100.19846481 | 0.852735 | 0.988545 | -2099.61218655 |  |
| **25-ts** | -2100.18277057 | 0.848066 | 0.98448 | -2099.59968941 | -182.93 |
| **26** | -660.169927221 | 0.24361 | 0.300667 | -659.998465387 |  |
| **27-ts** | -2100.080930 | 0.836210 | 0.976669 | -2099.434693 | -1533.05 |
| **6a** | -1917.47451596 | 0.566866 | 0.680011 | -1917.08730731 |  |
| **13a** | -2165.78242427 | 0.655182 | 0.777571 | -2165.31274404 |  |
| **14a-ts** | -2165.75459917 | 0.653546 | 0.776133 | -2165.28493196 | -760.15 |
| **15a** | -2165.77066912 | 0.653743 | 0.781075 | -2165.31399972 |  |
| **16a-ts** | -2577.64789186 | 0.842037 | 0.984619 | -2577.09246004 | -91.25 |
| **17a** | -2577.67734345 | 0.850076 | 0.987527 | -2577.12127666 |  |
| **18a-ts** | -2577.64643753 | 0.841918 | 0.982378 | -2577.09650672 | -250.81 |
| **6b** | -1270.64114633 | 0.56861 | 0.679564 | -1270.14727385 |  |
| **13b** | -1518.94981149 | 0.653285 | 0.777177 | -1518.36731963 |  |
| **14b-ts** | -1518.92785497 | 0.650565 | 0.775238 | -1518.34497707 | -751.56 |
| **15b** | -1518.95554606 | 0.657887 | 0.781035 | -1518.38671545 |  |
| **16b-ts** | -1930.82337276 | 0.840523 | 0.984215 | -1930.15625177 | -126.39 |
| **17b** | -1930.85288952 | 0.843276 | 0.985119 | -1930.18678769 |  |
| **18b-ts** | -1930.81750435 | 0.837863 | 0.981515 | -1930.15841347 | -263.01 |

^1^The electronic energy calculated by B3LYP in gas phase. ^2^The thermal correction to Gibbs free energy calculated by B3LYP in gas phase. ^3^The thermal correction to enthalpy calculated by B3LYP in gas phase. ^4^The electronic energy calculated by M11 in benzene. ^5^The B3LYP calculated imaginary frequencies for the transition states.

**7. B3LYP geometries for all the optimized compounds and transition states.**

**2**

B -0.00014700 1.94531900 0.00006300

H -0.00022400 3.13519400 0.00011700

O -1.08617700 1.20090400 -0.36634300

O 1.08598500 1.20101000 0.36641700

C -0.78921900 -0.19101900 -0.04520200

C 0.78926000 -0.19088600 0.04516100

C -1.48000800 -0.48437300 1.29334800

H -2.54923800 -0.27298100 1.19449100

H -1.36101600 -1.53228200 1.58820100

H -1.08444800 0.14891900 2.09365600

C 1.37945600 -1.07346000 1.14396900

H 1.13964800 -2.12859900 0.96880200

H 2.46930900 -0.97061700 1.14943000

H 1.01038900 -0.79137000 2.13268200

C 1.48018900 -0.48411700 -1.29334000

H 2.54938900 -0.27259400 -1.19441900

H 1.36134400 -1.53203900 -1.58820300

H 1.08461200 0.14913400 -2.09367100

C -1.37939800 -1.07349000 -1.14406400

H -1.13914400 -2.12858600 -0.96926500

H -2.46928900 -0.97105400 -1.14915800

H -1.01073700 -0.79095600 -2.13280700

**6**

C 1.28036900 -0.00003600 1.76026400

C -0.00000800 -0.00008500 2.35255700

C -1.28038600 -0.00004700 1.76023000

H -0.00001500 0.00015800 3.43565500

Mg 0.00001800 -0.00085800 -0.95289500

N 1.50190500 -0.00028500 0.44064400

C 2.46308200 0.00050800 2.71288900

H 3.09808100 -0.87636500 2.54664000

H 3.09666800 0.87854200 2.54724700

H 2.13430400 -0.00008400 3.75402700

C -2.46307400 0.00049400 2.71288400

H -2.13427600 -0.00022500 3.75401500

H -3.09659100 0.87859300 2.54735600

H -3.09814900 -0.87630300 2.54656900

N -1.50193000 -0.00030900 0.44066000

C 2.84948000 -0.00004200 -0.06449200

C 3.48801900 1.23015200 -0.35739100

C 3.48844000 -1.23008200 -0.35732300

C 4.76626800 1.20252700 -0.92832400

C 4.76672200 -1.20201300 -0.92827800

C 5.40781800 0.00038300 -1.21060200

H 5.26501100 2.13959800 -1.16163500

H 5.26578900 -2.13893500 -1.16156600

H 6.39973700 0.00055500 -1.65465700

C 2.80958200 2.57545300 -0.10517600

C 2.41140700 3.24891300 -1.43486200

C 3.67291300 3.52460300 0.74788700

H 1.88879200 2.38666200 0.45558000

H 1.76182100 2.60121600 -2.03533500

H 1.87987500 4.19030300 -1.24875600

H 3.29609400 3.47648300 -2.04129700

H 3.96135000 3.06194600 1.69868000

H 4.59281300 3.81670000 0.22813800

H 3.11800700 4.44311000 0.97343600

C 2.81053600 -2.57565600 -0.10499900

C 3.67407500 -3.52419300 0.74852300

C 2.41306500 -3.24959000 -1.43467100

H 1.88952100 -2.38714100 0.45543900

H 3.96202100 -3.06117400 1.69929000

H 3.11955000 -4.44291100 0.97412900

H 4.59427500 -3.81596800 0.22910400

H 1.76351500 -2.60224400 -2.03555400

H 3.29807800 -3.47702700 -2.04071100

H 1.88178900 -4.19110300 -1.24852300

C -2.84949100 -0.00004400 -0.06445000

C -3.48801400 1.23014800 -0.35731400

C -3.48844100 -1.23005300 -0.35734000

C -4.76624900 1.20256100 -0.92827300

C -4.76671400 -1.20197700 -0.92831400

C -5.40781200 0.00043000 -1.21060800

H -5.26498400 2.13964300 -1.16156600

H -5.26577800 -2.13889200 -1.16164800

H -6.39972600 0.00062400 -1.65468700

C -2.80957300 2.57543500 -0.10508700

C -3.67295800 3.52463700 0.74786600

C -2.41129700 3.24884700 -1.43477000

H -1.88882800 2.38667000 0.45574000

H -3.96148600 3.06202700 1.69865100

H -3.11805400 4.44314200 0.97342300

H -4.59280900 3.81673400 0.22802800

H -1.76166000 2.60113700 -2.03517000

H -3.29593800 3.47638600 -2.04128400

H -1.87978700 4.19025000 -1.24866200

C -2.81055900 -2.57564100 -0.10505800

C -2.41304100 -3.24951700 -1.43474400

C -3.67416600 -3.52421400 0.74836200

H -1.88957500 -2.38718300 0.45543600

H -1.76342500 -2.60217100 -2.03555300

H -1.88182000 -4.19106400 -1.24861600

H -3.29802700 -3.47688400 -2.04085100

H -3.96212300 -3.06126600 1.69915600

H -4.59436300 -3.81590400 0.22888600

H -3.11968900 -4.44297600 0.97390300

H -0.00000300 -0.00159900 -2.67317000

**12**

C -1.14277800 -0.72193200 -0.00021700

C -1.19895200 0.67333100 -0.00012000

C 0.00024700 1.38569300 0.00009400

C 1.19919100 0.67292000 0.00021600

C 1.14252200 -0.72232200 0.00012500

N -0.00025200 -1.42075700 -0.00009900

H 0.00041300 2.47254800 0.00016100

H -2.06016700 -1.30893200 -0.00037300

H -2.15797300 1.18299100 -0.00023100

H 2.15841100 1.18220800 0.00035900

H 2.05969800 -1.30964900 0.00018600

**13**

C 1.28596100 -0.57489700 -1.84946900

C 0.00002600 -0.55581300 -2.43751200

C -1.28579600 -0.57548300 -1.84916400

H -0.00004100 -0.70228400 -3.51089700

Mg 0.00023400 0.12505500 0.79486500

N 1.53699200 -0.37477800 -0.55309600

C 2.42798700 -0.90565900 -2.79900800

H 3.25863300 -0.20314900 -2.69442500

H 2.83247800 -1.89962400 -2.57542000

H 2.09153900 -0.90211200 -3.83802000

C -2.42770600 -0.90693300 -2.79860900

H -2.09115200 -0.90419100 -3.83758600

H -2.83222200 -1.90071100 -2.57431300

H -3.25836400 -0.20435200 -2.69470500

N -1.53680800 -0.37534400 -0.55286800

C 2.87066200 -0.59405200 -0.05568100

C 3.22085900 -1.87117000 0.45409300

C 3.81535600 0.46372200 -0.01552300

C 4.50893400 -2.06607800 0.96760100

C 5.09036000 0.21515300 0.50853600

C 5.44557200 -1.03843900 0.99389500

H 4.78062100 -3.04432800 1.35572400

H 5.81808300 1.02242400 0.53624900

H 6.44110000 -1.21134700 1.39436300

C 2.24200600 -3.04320700 0.47591500

C 1.94222300 -3.49101800 1.92031600

C 2.74064800 -4.23125000 -0.37097300

H 1.30023600 -2.70286300 0.03546300

H 1.52205500 -2.66792200 2.50737400

H 1.22014300 -4.31630100 1.92253200

H 2.84939000 -3.84313400 2.42618600

H 2.93102500 -3.93749200 -1.40960200

H 3.67117600 -4.65044600 0.03041100

H 1.99326300 -5.03386800 -0.37978300

C 3.49605500 1.87448400 -0.50529100

C 4.42191200 2.33094300 -1.65109600

C 3.55625200 2.88995700 0.65360300

H 2.47275500 1.86801700 -0.89073700

H 4.38143100 1.64910000 -2.50716300

H 4.13152700 3.32879500 -2.00307300

H 5.46656200 2.38830600 -1.32377200

H 2.90335200 2.59103400 1.47948800

H 4.57405000 2.97647800 1.05201800

H 3.24712600 3.88585600 0.31278600

C -2.87036100 -0.59490200 -0.05545500

C -3.21994100 -1.87174100 0.45536700

C -3.81560000 0.46235300 -0.01629400

C -4.50793300 -2.06696200 0.96889000

C -5.09057200 0.21353700 0.50776600

C -5.44517900 -1.03984000 0.99417000

H -4.77914200 -3.04502800 1.35780900

H -5.81873600 1.02044400 0.53468600

H -6.44066700 -1.21296600 1.39466100

C -2.24054900 -3.04329000 0.47805700

C -2.73824000 -4.23172200 -0.36885100

C -1.94129400 -3.49067700 1.92269300

H -1.29871200 -2.70264600 0.03801600

H -2.92799200 -3.93830200 -1.40767800

H -1.99059300 -5.03410900 -0.37694400

H -3.66890900 -4.65109600 0.03201500

H -1.52182400 -2.66726600 2.50980300

H -2.84851900 -3.84318200 2.42819200

H -1.21879100 -4.31558500 1.92544400

C -3.49692200 1.87295300 -0.50695100

C -3.55694300 2.88895800 0.65149300

C -4.42340000 2.32863500 -1.65254600

H -2.47379100 1.86666400 -0.89283600

H -2.90350200 2.59073100 1.47718400

H -3.24845700 3.88483700 0.31004100

H -4.57458000 2.97520400 1.05037500

H -4.38330800 1.64633500 -2.50826900

H -5.46790700 2.38611200 -1.32477300

H -4.13329900 3.32631500 -2.00523500

H 0.00070700 -0.21703400 2.51535800

C -0.00069700 3.12584500 -0.29402100

C -0.00112800 4.51731900 -0.24092100

C -0.00132200 5.13856000 1.00929900

C -0.00105700 4.34551900 2.15607300

C -0.00061300 2.95935400 2.01155000

N -0.00044500 2.35751100 0.80898300

H -0.00168700 6.22226900 1.08667000

H -0.00051000 2.59683700 -1.24309800

H -0.00131700 5.09550000 -1.15930600

H -0.00118300 4.78592300 3.14780700

H 0.00040000 2.28682100 2.86577600

**14-ts**

C 1.11505700 1.03169700 -2.13826400

C -0.19239100 1.32662500 -2.57629500

C -1.44395500 1.11184600 -1.95617800

H -0.24235900 1.85688400 -3.51942100

Mg -0.04757900 -0.46991400 0.15073200

N 1.41096100 0.38525600 -1.00385500

C 2.24050900 1.52787500 -3.03068300

H 2.73973100 2.39605100 -2.58564500

H 3.00685200 0.75894100 -3.16557100

H 1.86098900 1.82399100 -4.01107900

C -2.64674200 1.72344400 -2.65349800

H -3.38738400 0.95993400 -2.91009200

H -3.15126800 2.43790300 -1.99390600

H -2.35331900 2.24423000 -3.56708400

N -1.61777900 0.43358400 -0.81954700

C 2.77311600 0.28668600 -0.55682600

C 3.48696800 -0.92429300 -0.73568100

C 3.35790500 1.36837500 0.15335600

C 4.77493400 -1.03018100 -0.19453400

C 4.64369300 1.20399900 0.68276900

C 5.35248900 0.01744200 0.51464200

H 5.33292200 -1.95290600 -0.33072700

H 5.09910000 2.02067500 1.23679000

H 6.35061900 -0.08761700 0.93175500

C 2.91028900 -2.10544400 -1.51493000

C 2.85921200 -3.39294400 -0.66974200

C 3.68766600 -2.35032200 -2.82473200

H 1.88105400 -1.85395800 -1.79134900

H 2.28780400 -3.24504800 0.25213800

H 2.38181400 -4.19993900 -1.23839100

H 3.86438600 -3.73293900 -0.39274400

H 3.69339900 -1.46117500 -3.46520600

H 4.73113900 -2.62077100 -2.62319000

H 3.23312200 -3.17162400 -3.39174700

C 2.62870800 2.69568900 0.37083300

C 3.48219300 3.90907700 -0.04978400

C 2.15912800 2.85559100 1.83100300

H 1.73213100 2.69773000 -0.25496800

H 3.84420300 3.81336600 -1.07966900

H 2.89009700 4.82944800 0.01781900

H 4.35772200 4.03549600 0.59735900

H 1.45951900 2.06323100 2.11744400

H 3.00707800 2.82548500 2.52596100

H 1.64843100 3.81680400 1.96769400

C -2.92877800 0.28151900 -0.24779800

C -3.73926100 -0.82544500 -0.61112400

C -3.35961100 1.18736700 0.75511300

C -4.96240600 -1.00338400 0.04831200

C -4.59258800 0.96216500 1.38008600

C -5.39179300 -0.12381300 1.03727500

H -5.58997800 -1.84971300 -0.21903700

H -4.92970900 1.65031700 2.15103300

H -6.34410900 -0.28296900 1.53630400

C -3.33480000 -1.82863600 -1.69229100

C -4.35907900 -1.87733700 -2.84489900

C -3.12261900 -3.24130100 -1.11196800

H -2.37897100 -1.50348300 -2.11409900

H -4.52732500 -0.88893700 -3.28664700

H -4.00558600 -2.54581100 -3.63898800

H -5.33036400 -2.25431500 -2.50364600

H -2.33477800 -3.24065400 -0.35202000

H -4.04240700 -3.62889000 -0.65730800

H -2.82421200 -3.93809100 -1.90497300

C -2.52745800 2.39830100 1.17477700

C -2.07206900 2.29538400 2.64436800

C -3.27947800 3.72228600 0.93084300

H -1.62667900 2.41652400 0.55345600

H -1.48176000 1.38900900 2.81517300

H -1.45401200 3.16117800 2.91306300

H -2.92992400 2.27594900 3.32748300

H -3.59579200 3.82239200 -0.11372800

H -4.17670200 3.79655100 1.55656400

H -2.63598000 4.57672300 1.17271000

H -0.40976800 -2.30192500 0.54154500

C 1.33505400 -0.92417300 2.80687900

C 1.75750200 -2.08445000 3.42829900

C 0.83762600 -3.16323200 3.50498800

C -0.37448500 -3.07939800 2.87370300

C -0.65589200 -1.92179000 2.05830800

N 0.12677200 -0.76712600 2.21822300

H 1.08461400 -4.04220900 4.09682200

H 1.97227600 -0.04289800 2.80234100

H 2.72880900 -2.12923900 3.90754400

H -1.10764500 -3.87795800 2.93000100

H -1.70839000 -1.71413200 1.86973300

**15**

C 1.26250500 -1.40126000 1.91934100

C -0.02316300 -1.73352400 2.39598200

C -1.30381600 -1.40491700 1.90192300

H -0.02825900 -2.35450300 3.28309000

Mg -0.00725800 0.13307400 -0.27442600

N 1.49972400 -0.64921700 0.83755700

C 2.43460200 -1.96241100 2.70423600

H 3.01934600 -2.65339800 2.08661200

H 3.11867200 -1.16549700 3.01314700

H 2.09591400 -2.49740100 3.59355900

C -2.48417900 -1.98174500 2.66284400

H -2.15561900 -2.52472500 3.55111700

H -3.17861300 -1.19319500 2.96988000

H -3.05439200 -2.66946500 2.02807200

N -1.52801400 -0.64402400 0.82421700

C 2.85043000 -0.37706400 0.41916400

C 3.48728900 0.81560300 0.84185200

C 3.49025000 -1.25299000 -0.49311200

C 4.76486400 1.10347500 0.34494600

C 4.76531000 -0.91504500 -0.96253700

C 5.40402200 0.25087200 -0.54968700

H 5.26294900 2.01592600 0.66181600

H 5.26453000 -1.57458200 -1.66729200

H 6.39351300 0.49513600 -0.92673000

C 2.82556500 1.79868900 1.80628800

C 2.55333200 3.15959000 1.13268300

C 3.65160900 1.98886500 3.09434100

H 1.85918000 1.37802600 2.10266600

H 1.93975600 3.05586800 0.23026000

H 2.03032900 3.83024300 1.82542300

H 3.48881800 3.65098400 0.83928100

H 3.82943900 1.03620100 3.60614200

H 4.62868000 2.43887300 2.88302200

H 3.12499200 2.65305500 3.78995900

C 2.81693700 -2.52718000 -1.00114100

C 3.70630200 -3.77454600 -0.83569700

C 2.36969800 -2.37076900 -2.46952000

H 1.91670600 -2.69365600 -0.40170900

H 4.03263900 -3.90217100 0.20282600

H 3.15466600 -4.67510800 -1.13031100

H 4.60482700 -3.72190600 -1.46110800

H 1.69005800 -1.52023000 -2.59833600

H 3.23031700 -2.20391800 -3.12806400

H 1.85217800 -3.27420700 -2.81462500

C -2.87443900 -0.37901300 0.38711300

C -3.53527500 0.79547200 0.82384000

C -3.48631100 -1.24331500 -0.55475700

C -4.81267300 1.07086900 0.31971900

C -4.76199800 -0.91786600 -1.03145500

C -5.42742400 0.22555700 -0.59872700

H -5.32935100 1.96875300 0.64815200

H -5.23996000 -1.56918600 -1.75822500

H -6.41741800 0.45974600 -0.98080700

C -2.89305200 1.77942300 1.80015300

C -3.72697900 1.95333600 3.08502300

C -2.63658300 3.14653500 1.13217200

H -1.92248900 1.37001800 2.09846500

H -3.89129400 0.99686900 3.59420300

H -3.21398200 2.62438100 3.78426700

H -4.71034400 2.38805200 2.87088900

H -2.04275300 3.05010100 0.21633800

H -3.57922000 3.63633300 0.85981400

H -2.10289800 3.81414400 1.81950700

C -2.78113500 -2.48813800 -1.09093800

C -2.33127600 -2.28071800 -2.55260000

C -3.64115700 -3.76005100 -0.96231800

H -1.88047900 -2.64893500 -0.49032400

H -1.67923700 -1.40533700 -2.65662500

H -1.78439100 -3.15920100 -2.91662900

H -3.19416100 -2.12615700 -3.21132800

H -3.96562900 -3.92463100 0.07163700

H -4.53982600 -3.71067800 -1.58782800

H -3.06816100 -4.63874500 -1.28133700

C -1.16426400 1.86180000 -2.35233500

C -1.13665800 3.05362200 -3.02739100

H -2.11734900 1.42356800 -2.05077100

C 1.27140800 3.10714300 -2.78501500

C 0.12449600 3.76467300 -3.06528200

H -2.06195700 3.53137200 -3.33154500

H 2.23156100 3.60876100 -2.69574400

H 0.11897600 4.84095900 -3.23236500

N -0.03772500 1.20163500 -1.92356200

C 1.17846200 1.60380800 -2.66318800

H 1.13844900 1.14544900 -3.67419300

H 2.06029800 1.18571100 -2.16413400

**16-ts**

C 2.15713600 -1.71392100 -1.86164700

C 1.13066100 -2.61041700 -2.21792000

C -0.17145500 -2.79117000 -1.70500600

H 1.42358000 -3.35180100 -2.95333100

Mg 0.21104200 -0.31475900 -0.10779800

N 2.02869500 -0.70890400 -0.98513600

C 3.50390300 -2.00312900 -2.50665000

H 4.12957000 -1.11318700 -2.58376000

H 4.05554600 -2.73927600 -1.90935700

H 3.36506000 -2.43044100 -3.50345200

C -0.89302600 -4.01697700 -2.24523500

H -1.32484200 -3.79292300 -3.22771700

H -0.19018700 -4.84364800 -2.38057500

H -1.70369900 -4.34449800 -1.59316600

N -0.76583500 -1.97910600 -0.82375900

C 3.19291400 0.03709100 -0.57839200

C 3.48418300 1.28449000 -1.18875400

C 4.00094900 -0.45244500 0.48076800

C 4.57634600 2.02163400 -0.71301600

C 5.07308400 0.33268600 0.92310300

C 5.36307900 1.56097500 0.33794100

H 4.81445900 2.97487100 -1.17634500

H 5.69340500 -0.02843100 1.73923600

H 6.20104800 2.15372500 0.69520100

C 2.68371800 1.82614500 -2.37391200

C 3.46766000 1.67367400 -3.69516500

C 2.25414100 3.29399400 -2.18856800

H 1.77349800 1.22408500 -2.46417000

H 3.72251200 0.62997100 -3.90455400

H 2.87353600 2.04943500 -4.53707800

H 4.40308400 2.24570400 -3.66380400

H 1.68524400 3.44506400 -1.26760700

H 3.11807800 3.96914100 -2.17305700

H 1.61920300 3.60278000 -3.02797700

C 3.75617400 -1.80652200 1.14844200

C 3.35266400 -1.66136100 2.62926100

C 4.98287700 -2.73421000 1.02522400

H 2.92517100 -2.29430500 0.63025700

H 2.45156100 -1.05200600 2.75387400

H 3.15968100 -2.64810900 3.06845200

H 4.15264700 -1.19054700 3.21373100

H 5.30469000 -2.84933600 -0.01536100

H 5.83702800 -2.34680400 1.59298200

H 4.74837000 -3.72927300 1.42194300

C -2.06285600 -2.30227200 -0.28408200

C -2.12960200 -2.87487300 1.01419600

C -3.24901100 -1.99371900 -0.99636200

C -3.38852400 -3.11879000 1.57524800

C -4.48190800 -2.25466300 -0.38305400

C -4.55949100 -2.80882700 0.88957400

H -3.45368900 -3.55927900 2.56530900

H -5.39831800 -2.01877900 -0.91748100

H -5.52734200 -3.00230300 1.34468600

C -0.87093400 -3.28970500 1.77708700

C -0.53424900 -4.77247600 1.51177200

C -0.94640500 -3.02024700 3.29022700

H -0.03256000 -2.70026000 1.38762100

H -0.38728600 -4.97153400 0.44543900

H 0.38376600 -5.05927600 2.03922900

H -1.34409700 -5.42213400 1.86572300

H -1.21702900 -1.98076800 3.49935300

H -1.67182800 -3.67536200 3.78750900

H 0.02991200 -3.21548600 3.74883600

C -3.24552600 -1.40824700 -2.40849800

C -3.90615800 -0.01643600 -2.45694900

C -3.93673700 -2.34753500 -3.41974300

H -2.20372100 -1.28754300 -2.72217400

H -3.43119600 0.68009200 -1.76165300

H -3.83602500 0.39961700 -3.47005200

H -4.97113300 -0.07815500 -2.20025200

H -3.51233200 -3.35631100 -3.40522000

H -5.00823800 -2.43858900 -3.20612200

H -3.83733400 -1.94978500 -4.43704500

H -0.36841200 1.48362900 -1.12462500

B -0.97279500 2.23141400 -0.38594400

O -0.48295600 3.48870300 -0.12890800

O -2.34630700 2.14681000 -0.29782700

C -1.58283900 4.31964600 0.34750100

C -2.86886100 3.48960000 -0.08471200

C -1.43739600 4.49452200 1.86429300

H -0.45966100 4.93780400 2.07833700

H -2.20721100 5.16990900 2.25428900

H -1.50139400 3.54512400 2.40072200

C -3.97092700 3.41024000 0.97260700

H -4.37659900 4.40432000 1.19297500

H -4.78933900 2.78729700 0.59716500

H -3.60638400 2.96604700 1.90099800

C -3.46814400 3.93632800 -1.42626800

H -4.25143300 3.23077700 -1.72001500

H -3.91443900 4.93413000 -1.35762600

H -2.71306100 3.94706700 -2.21825300

C -1.44012100 5.68469800 -0.33123600

H -2.26454800 6.35087200 -0.05268200

H -0.50476100 6.15156700 -0.00652400

H -1.41198900 5.59831500 -1.41969700

C 1.14426300 1.24927800 3.50599500

C -1.24412100 0.86798400 3.63204900

C 0.06343100 0.96654600 4.25899200

H 2.15062200 1.26188300 3.91649500

H -2.13976000 0.71607300 4.22566500

H 0.16141600 0.73666900 5.31854700

N -0.20754800 0.91884900 1.43464900

C -1.29417500 0.75217800 2.27299000

H -2.22044700 0.46726200 1.77583200

C 0.92946300 1.64929200 2.06285500

H 0.73580500 2.73356900 1.99648300

H 1.83514100 1.47504800 1.47102400

**17**

C -1.74264400 -1.22009600 -1.98354200

C -0.53156700 -1.38965900 -2.69991000

C 0.81787500 -1.35128100 -2.29767100

H -0.67541000 -1.82171700 -3.68439000

Mg -0.12251100 0.40744700 -0.17924100

N -1.86753300 -0.57475600 -0.82086000

C -2.92469000 -1.93104000 -2.63210200

H -2.82304300 -3.01285700 -2.48288300

H -3.88840300 -1.62711800 -2.22768700

H -2.91920600 -1.75735300 -3.71273300

C 1.77091300 -1.96796700 -3.31659400

H 1.99635400 -1.22389200 -4.09004500

H 2.71339300 -2.28809800 -2.87291700

H 1.31027300 -2.82403100 -3.81620200

N 1.27838000 -0.83804000 -1.14179700

C -3.14131300 -0.63701700 -0.13268500

C -3.38661000 -1.68601200 0.78964800

C -4.14833500 0.33465100 -0.37904300

C -4.62914400 -1.75149100 1.43342200

C -5.37630200 0.21292100 0.28537400

C -5.62567000 -0.81707900 1.18470300

H -4.81929400 -2.55886600 2.13550700

H -6.15580100 0.94304000 0.09071400

H -6.58710500 -0.88876300 1.68630800

C -2.36581400 -2.77709800 1.09979200

C -2.84465900 -4.16082800 0.61401100

C -2.02786300 -2.82608400 2.60210700

H -1.44621400 -2.54177900 0.55805800

H -3.07201000 -4.15571900 -0.45699200

H -2.07385300 -4.92030700 0.79371200

H -3.75159600 -4.47602600 1.14335300

H -1.64772400 -1.86198700 2.95434700

H -2.90799700 -3.07493200 3.20591400

H -1.26609900 -3.58741800 2.80314100

C -3.97094100 1.49667800 -1.35512400

C -4.51534400 2.82135900 -0.78286900

C -4.63683500 1.23088700 -2.72245200

H -2.90061300 1.62717100 -1.53435400

H -4.16406100 3.00025200 0.23855400

H -4.19694300 3.66073500 -1.41128400

H -5.61122900 2.83564200 -0.76360000

H -4.20279900 0.36888400 -3.23485500

H -5.71226900 1.04737600 -2.60604700

H -4.51428400 2.10242100 -3.37704600

C 2.60305400 -1.22224200 -0.69318000

C 3.76575600 -0.47443700 -1.02012700

C 2.73027600 -2.39331500 0.10949500

C 5.00909100 -0.90151000 -0.52987600

C 3.99738100 -2.77173400 0.56912800

C 5.13523700 -2.03494400 0.25987000

H 5.89854800 -0.33293200 -0.78570400

H 4.09784500 -3.66413600 1.17805000

H 6.10969800 -2.34660200 0.62643900

C 3.75968500 0.75358300 -1.92688400

C 4.42548700 0.46593900 -3.29132000

C 4.47680300 1.95910200 -1.28194300

H 2.71678600 1.02406000 -2.11440100

H 3.96099600 -0.37363500 -3.81314700

H 4.35916900 1.34756300 -3.94094700

H 5.48842400 0.22853100 -3.16335200

H 4.12035600 2.16406500 -0.26911100

H 5.55762400 1.78945500 -1.21918000

H 4.33015800 2.86221200 -1.88585700

C 1.53508200 -3.28670600 0.44097400

C 1.63114000 -3.95483900 1.82519100

C 1.33075000 -4.37939300 -0.63091800

H 0.64261700 -2.65335500 0.43759300

H 1.84900500 -3.23165200 2.61709600

H 0.68366400 -4.44952100 2.06471500

H 2.40632700 -4.72932500 1.85331500

H 1.13462800 -3.95559000 -1.61848400

H 2.22106000 -5.01583000 -0.70502100

H 0.47975800 -5.01943000 -0.36695000

H -1.36542300 2.71858300 1.73474000

B -0.20388500 2.50471200 1.43603100

O 0.77324600 3.22036500 2.21327400

O 0.16155100 1.02909800 1.68503200

C 1.49783200 2.35609100 3.08823000

C 0.70343300 0.98715000 3.02794900

C 2.94075900 2.22412800 2.57031800

H 3.34457700 3.22863400 2.40791000

H 3.59042800 1.70338300 3.28323800

H 2.98104400 1.68428500 1.62032300

C 1.55917900 -0.26528200 3.19780000

H 2.06033100 -0.25612500 4.17265900

H 0.92490900 -1.15739500 3.16089300

H 2.32081900 -0.35565100 2.41980400

C -0.47690700 0.94258100 4.01170100

H -1.10320600 0.07452700 3.78413700

H -0.12647100 0.84895400 5.04480200

H -1.10080200 1.83573800 3.93371700

C 1.53152200 2.99348100 4.48428500

H 2.01843800 2.33861000 5.21750800

H 2.09933600 3.92889400 4.43825600

H 0.52617900 3.23321900 4.83799500

C -1.14986500 3.40602800 -0.73174000

C 1.22326200 3.25085100 -2.15571800

C -1.08455500 3.91813200 -1.97982300

H -2.03780300 3.49144900 -0.11969900

C 0.10804400 3.67810400 -2.77520900

H 2.14407800 3.07177200 -2.70083200

H -1.93544700 4.44755200 -2.39532700

H 0.08073300 3.83437200 -3.85117100

N -0.09347900 2.67281400 -0.14165800

C 1.22413400 3.15046300 -0.64613400

H 1.99521900 2.46751000 -0.29093800

H 1.44614000 4.13196100 -0.19197300

**18-ts**

C 1.97050600 -2.53854700 0.81634600

C 0.85175200 -3.39079300 0.72560900

C -0.48334300 -3.14009800 0.35683800

H 1.05639900 -4.42115300 0.99096200

N 1.98486200 -1.24262900 0.47832300

C 3.21070200 -3.20480000 1.40118700

H 3.22749800 -4.27216000 1.16636000

H 4.14276100 -2.75298600 1.06191300

H 3.18008600 -3.11178300 2.49392200

C -1.38321900 -4.36573300 0.31149700

H -0.79927300 -5.28356600 0.40490800

H -2.11729800 -4.34300100 1.12355500

H -1.95222400 -4.40615200 -0.62148900

N -0.99065600 -1.93165900 0.08261200

C 3.24623500 -0.54856600 0.49323200

C 4.03592900 -0.53269500 -0.69181200

C 3.68132900 0.13902400 1.65454300

C 5.24176600 0.17903300 -0.68163000

C 4.89445700 0.84025900 1.60278900

C 5.67219800 0.86514500 0.45155500

H 5.85867800 0.19807900 -1.57472300

H 5.23605600 1.37047700 2.48782600

H 6.61159900 1.41169100 0.43541300

C 3.61643700 -1.29069900 -1.95653400

C 4.11561500 -2.75249000 -1.96325300

C 4.07941200 -0.61263900 -3.26106100

H 2.52081900 -1.32784200 -1.96492900

H 3.67856200 -3.34257600 -1.15580700

H 3.84806100 -3.23798000 -2.90988400

H 5.20741400 -2.79052900 -1.86318300

H 3.85713700 0.45835500 -3.27770900

H 5.15806400 -0.73147900 -3.41823900

H 3.57949100 -1.07828200 -4.11821400

C 2.90647100 0.12310700 2.97297000

C 2.46561100 1.53378600 3.41023800

C 3.72382200 -0.54505400 4.09898400

H 1.99832100 -0.46882200 2.82478200

H 1.80316200 1.99020200 2.67048200

H 1.91574800 1.47755700 4.35749100

H 3.32864300 2.19274600 3.56613200

H 4.06199700 -1.54900300 3.82190200

H 4.61432500 0.04411200 4.34941300

H 3.11740800 -0.62884000 5.00870400

C -2.39765800 -1.83957700 -0.20602900

C -3.33580600 -1.75077800 0.85567100

C -2.83525300 -1.79937300 -1.55402700

C -4.69106300 -1.59915200 0.53757700

C -4.20368800 -1.65203300 -1.81548800

C -5.12992700 -1.54809500 -0.78232500

H -5.41728800 -1.51933800 1.34157300

H -4.54901400 -1.62582200 -2.84591000

H -6.18800000 -1.43492700 -1.00398300

C -2.92560400 -1.81791200 2.32836000

C -3.73708800 -2.86944300 3.11307100

C -3.03243800 -0.44295200 3.01806900

H -1.87535700 -2.12070300 2.37261500

H -3.70729200 -3.85527000 2.63483900

H -3.33413700 -2.97306400 4.12742500

H -4.79037500 -2.58142900 3.20944600

H -2.36893600 0.28963900 2.55008900

H -4.05561200 -0.05251000 2.96973300

H -2.75091400 -0.52868900 4.07524900

C -1.86782400 -1.94386300 -2.72788600

C -1.94875500 -0.74194300 -3.68696000

C -2.09359800 -3.25903500 -3.50121200

H -0.85325200 -1.97486100 -2.31824200

H -1.79566900 0.19670300 -3.14661000

H -1.18458900 -0.82246000 -4.47035200

H -2.92396600 -0.68534400 -4.18479600

H -1.98214700 -4.13500800 -2.85349300

H -3.09799500 -3.29541500 -3.93975200

H -1.36877700 -3.35239000 -4.31930000

H -0.17779200 1.06259100 1.55723500

B -0.83800700 2.50346800 0.07141500

O 0.03686100 3.56935300 0.15625200

O -0.42947300 1.58181600 -0.95668600

C 1.00341500 3.48069700 -0.91432600

C 0.34093500 2.43058400 -1.89313000

C 1.16053700 4.88063100 -1.51611700

H 1.60313300 5.54570600 -0.76797200

H 1.82563000 4.86347100 -2.38753900

H 0.19937300 5.30488700 -1.81432100

C -0.67225400 3.06494500 -2.85550300

H -0.16846400 3.69178400 -3.59822600

H -1.21279600 2.27687900 -3.38658200

H -1.40467000 3.67940700 -2.32369000

C 1.32146600 1.55653500 -2.65639400

H 0.78480200 0.83245900 -3.27814100

H 1.93995400 2.17249000 -3.31883500

H 1.98716400 1.01630100 -1.98196300

C 2.34048200 3.03012300 -0.32019800

H 3.13811300 3.02151700 -1.07028200

H 2.62288700 3.73125700 0.47092500

H 2.28288300 2.03788900 0.13035300

Mg 0.14558300 -0.19276400 0.36185800

C -3.23939800 1.85309200 0.11190800

C -4.52894800 2.19119000 0.34606800

H -2.97262800 0.99291700 -0.49159400

C -3.83009400 4.17231700 1.56076000

C -4.81862500 3.46461200 0.98762600

H -5.32369700 1.56178700 -0.03586000

H -4.00235800 5.15165100 1.99939900

H -5.83270900 3.85660400 0.94734300

C -2.44330900 3.58205100 1.65843700

H -1.67571300 4.35965000 1.62530200

H -2.31359400 3.04928000 2.61930300

N -2.17889000 2.63808900 0.54970000

**19**

B -0.13889000 0.03435800 -0.10136800

O 0.62085900 1.14369400 0.20904400

O 0.60893700 -1.10639000 -0.30577500

C 2.00851700 0.77711000 -0.02199100

C 1.96630400 -0.79673100 0.11443400

C 2.36792600 1.25314300 -1.43654100

H 2.17052700 2.32723500 -1.50946300

H 3.42464700 1.08121800 -1.66735700

H 1.76108400 0.74621400 -2.19371700

C 2.93518400 -1.55355000 -0.79225200

H 3.97412200 -1.29810100 -0.55376100

H 2.80987800 -2.63132900 -0.64559800

H 2.75481000 -1.33460100 -1.84717900

C 2.10834300 -1.28168800 1.56374600

H 1.86541200 -2.34805600 1.60668700

H 3.12840500 -1.14510400 1.93794400

H 1.42006500 -0.75078900 2.22917100

C 2.87476500 1.49568100 1.01095500

H 3.92553400 1.19873000 0.91476700

H 2.81344400 2.57737400 0.85291500

H 2.54377500 1.28563600 2.03052100

C -2.27738700 1.24964400 -0.06947400

C -3.61061000 1.25580700 0.14901700

H -1.67795700 2.15394000 -0.09145300

C -3.67087800 -1.17211400 0.08826700

C -4.29302800 -0.01364600 0.36520500

H -4.13704700 2.19674100 0.26301500

H -4.12987800 -2.13569900 0.29212800

H -5.28734500 -0.00711100 0.80609000

C -2.31674300 -1.15016200 -0.58301200

H -1.71594300 -2.02118500 -0.30884100

H -2.43702200 -1.18249300 -1.68239200

N -1.55782600 0.06231400 -0.21184500

**20-ts**

C 2.56589600 -2.27346900 -0.47577300

C 1.61294200 -3.14595900 -1.05566600

C 0.21446500 -3.03621500 -1.16567100

H 2.01463200 -4.10817300 -1.35242900

Mg 0.47287000 -0.17034600 -0.50273000

N 2.31517300 -1.01306700 -0.12449900

C 3.92596700 -2.90227300 -0.21599500

H 4.71636400 -2.16191800 -0.09320900

H 3.88245900 -3.49799800 0.70470400

H 4.19542200 -3.58328400 -1.02854600

C -0.50703200 -4.29490000 -1.62036300

H 0.19177800 -4.99843600 -2.07860200

H -0.98631500 -4.79918800 -0.77419400

H -1.29868200 -4.06256000 -2.33773800

N -0.49908900 -1.93804600 -0.86366200

C 3.33518600 -0.19546600 0.47778000

C 3.40596000 -0.09567000 1.89010600

C 4.20706100 0.56748500 -0.34350100

C 4.36629800 0.75319200 2.45564700

C 5.15314900 1.39326100 0.27697800

C 5.23893600 1.49070600 1.66263800

H 4.43201700 0.83280400 3.53750400

H 5.83301700 1.97457700 -0.33970900

H 5.98003800 2.14023900 2.12099700

C 2.49056800 -0.89574200 2.81555600

C 1.66587400 0.01389500 3.74817400

C 3.28789300 -1.92858300 3.63861900

H 1.78496000 -1.45248600 2.19077900

H 1.03935400 0.71574100 3.18776700

H 1.00414000 -0.59059300 4.37964700

H 2.31149400 0.59947800 4.41311400

H 3.86698400 -2.59800600 2.99306700

H 3.99115100 -1.43582200 4.32041800

H 2.61067300 -2.54181800 4.24519400

C 4.16630600 0.50709500 -1.87136000

C 5.37837600 -0.25750000 -2.44493700

C 4.08216700 1.90539700 -2.51399800

H 3.26280200 -0.03934200 -2.15837800

H 5.43636500 -1.28302300 -2.06731200

H 5.31608900 -0.30525100 -3.53895200

H 6.31770200 0.24640800 -2.18630000

H 3.24176000 2.48171700 -2.11851500

H 5.00013100 2.48225100 -2.34897300

H 3.94943100 1.81045400 -3.59838200

C -1.92622900 -2.05951500 -0.70814900

C -2.44845600 -2.56992200 0.51030100

C -2.80133400 -1.61776000 -1.72931700

C -3.83760100 -2.63540500 0.67042600

C -4.18343700 -1.71809000 -1.52256500

C -4.70554200 -2.22110000 -0.33606500

H -4.24720800 -3.02097600 1.60042000

H -4.86181000 -1.39286800 -2.30675200

H -5.78112700 -2.28980900 -0.19543900

C -1.55393000 -3.02987300 1.66272900

C -1.86279900 -4.47331800 2.10958000

C -1.65309800 -2.07168600 2.86697600

H -0.51677000 -3.01198900 1.31720000

H -1.80605600 -5.18156400 1.27545800

H -1.14540500 -4.79459000 2.87420600

H -2.86524700 -4.55684600 2.54536300

H -1.38906000 -1.04581200 2.59200700

H -2.67074000 -2.05425400 3.27503300

H -0.97831200 -2.39437600 3.66972300

C -2.29779100 -1.03876900 -3.04925300

C -2.85392600 0.37839800 -3.29318200

C -2.61923700 -1.95925400 -4.24407200

H -1.20591300 -0.96536800 -2.98468700

H -2.63855700 1.03978700 -2.44849200

H -2.41393300 0.81209900 -4.19980100

H -3.94068600 0.35807200 -3.43801900

H -2.18307800 -2.95639200 -4.11862900

H -3.70166900 -2.08316500 -4.36810000

H -2.22389700 -1.53303400 -5.17419300

H -0.44242100 0.72889700 1.00697500

C 0.19601000 2.08451600 -2.57195700

C 0.09597100 3.39145200 -2.98723300

C 0.55369800 4.40352700 -2.09679500

C 0.98998300 4.07027000 -0.84605500

C 0.89101100 2.69212000 -0.39182200

N 0.66426600 1.69653900 -1.35790600

H 0.59430500 5.43828600 -2.43003200

H -0.05341600 1.27377400 -3.25312400

H -0.24807100 3.62676700 -3.98784900

H 1.37003100 4.81713400 -0.15553000

H 1.61522500 2.38932800 0.37049700

O -2.25548900 1.73746500 0.12788500

O -1.57006300 2.21130000 2.29651000

C -3.25007800 2.63405100 0.67837600

C -2.98710500 2.50695400 2.23145100

C -2.96982200 4.04366500 0.13126900

H -2.96067000 4.00324000 -0.96257600

H -3.73702500 4.76046100 0.44491000

H -1.99375300 4.41624200 0.45674200

C 3.25056600 3.77994200 3.03747400

H -4.30333300 4.07942300 2.96965500

H -3.01785300 3.59995600 4.09246600

H -2.62820800 4.60958800 2.69399700

C -3.72949400 1.32323200 2.86876500

H -4.80629500 1.51254300 2.94587300

H -3.57828800 0.40574900 2.29305200

C -4.62993400 2.16428400 0.21690000

H -5.42291500 2.75311000 0.69343500

H -4.71895300 2.29450300 -0.86689400

H -4.79033700 1.10732400 0.44100500

B -1.17287800 1.75851800 1.02989600

H -0.17210700 2.61254800 0.49687100

H -3.33613700 1.16206200 3.87780000

**21-ts**

C -1.28154600 0.47611800 2.18776400

C -0.00003000 0.57480300 2.77019500

C 1.28150500 0.47614300 2.18776300

H -0.00003800 0.84976600 3.81793100

Mg -0.00001100 -0.52621100 -0.33216100

N -1.51321600 0.12055700 0.92029600

C -2.44801300 0.84076500 3.09057200

H -2.87742500 1.80421500 2.79284400

H -3.25277100 0.10369600 3.02226900

H -2.12979400 0.92017400 4.13230200

C 2.44789200 0.84095100 3.09060400

H 2.12978600 0.91944900 4.13243900

H 3.25316400 0.10452000 3.02167300

H 2.87654400 1.80492600 2.79347900

N 1.51319400 0.12056900 0.92034000

C -2.84697000 0.16226400 0.38504600

C -3.63671800 -1.01566800 0.36284900

C -3.32527500 1.35767900 -0.21238900

C -4.88188000 -0.97754300 -0.27753600

C -4.57554400 1.33783600 -0.84386100

C -5.35126000 0.18284300 -0.88508000

H -5.49382800 -1.87573100 -0.29942800

H -4.94801700 2.24619500 -1.31058000

H -6.31846500 0.18956600 -1.38090100

C -3.18664500 -2.31649700 1.02755700

C -3.04740500 -3.46698900 0.01186300

C -4.13282900 -2.72980700 2.17376300

H -2.19735500 -2.14532100 1.46279400

H -2.32964200 -3.21299000 -0.77360100

H -2.69326400 -4.37615700 0.51300000

H -4.00786400 -3.70204400 -0.46296800

H -4.22873600 -1.94144100 2.92844500

H -5.13915200 -2.95476700 1.80075400

H -3.75492000 -3.63007700 2.67308700

C -2.53290500 2.66543700 -0.18490800

C -3.32076200 3.79613200 0.50838600

C -2.09619700 3.10908700 -1.59595000

H -1.62311200 2.49468300 0.39773200

H -3.64889300 3.50842900 1.51348800

H -2.69667000 4.69313900 0.59980600

H -4.21484800 4.07373400 -0.06247200

H -1.44123000 2.36886300 -2.06598000

H -2.96296900 3.26782000 -2.24912900

H -1.54529600 4.05637700 -1.54235800

C 2.84693300 0.16224900 0.38510000

C 3.63663200 -1.01570300 0.36289200

C 3.32527200 1.35763800 -0.21232800

C 4.88181300 -0.97761700 -0.27745600

C 4.57554600 1.33776900 -0.84378400

C 5.35124300 0.18276000 -0.88498900

H 5.49373500 -1.87582400 -0.29934400

H 4.94804900 2.24611600 -1.31050600

H 6.31846000 0.18945800 -1.38079300

C 3.18650000 -2.31653200 1.02755500

C 4.13244100 -2.72970500 2.17401600

C 3.04760000 -3.46710100 0.01189900

H 2.19709700 -2.14542500 1.46254900

H 4.22790600 -1.94138900 2.92880300

H 3.75461200 -3.63011900 2.67314000

H 5.13893600 -2.95436700 1.80128800

H 2.33010600 -3.21316800 -0.77382900

H 4.00821500 -3.70221400 -0.46258700

H 2.69328100 -4.37622300 0.51299100

C 2.53293400 2.66541500 -0.18488500

C 2.09640800 3.10914300 -1.59596100

C 3.32074500 3.79605600 0.50855100

H 1.62306500 2.49466900 0.39763100

H 1.44152700 2.36893100 -2.06612500

H 1.54547000 4.05641300 -1.54237900

H 2.96325900 3.26795100 -2.24901600

H 3.64857200 3.50837200 1.51375600

H 4.21501900 4.07350800 -0.06208400

H 2.69674400 4.69314900 0.59974800

H 0.00006500 -2.31207000 -1.07873400

C -1.16162300 -0.48950100 -2.86442800

C -1.20459200 -1.83781500 -3.09526300

C 0.00007200 -2.54250000 -2.75454700

C 1.20475900 -1.83785900 -3.09525600

C 1.16184100 -0.48954800 -2.86440400

N 0.00012100 0.15248100 -2.49133800

H 0.00004900 -3.62889200 -2.76505500

H -2.05579900 0.12945900 -2.87471300

H -2.13313500 -2.35479000 -3.31198700

H 2.13328500 -2.35486700 -3.31197300

H 2.05604300 0.12937000 -2.87466400

**22**

C 1.28357600 -2.33698100 0.59493100

C 0.00089500 -2.90775400 0.73826200

C -1.28211000 -2.33722300 0.59668700

H 0.00119300 -3.95772800 1.00339000

Mg 0.00014900 0.24941600 -0.07268900

N 1.51247700 -1.05794800 0.27250400

C 2.46252300 -3.26459500 0.82685300

H 3.07927600 -3.34214300 -0.07523500

H 3.11470700 -2.87796600 1.61714400

H 2.13029600 -4.26601300 1.10716800

C -2.46049000 -3.26486300 0.83118500

H -2.12765000 -4.26566700 1.11297700

H -3.11210200 -2.87705200 1.62132200

H -3.07801400 -3.34426500 -0.07020400

N -1.51158600 -1.05847000 0.27370500

C 2.86051400 -0.56618200 0.15086900

C 3.48539900 0.04141100 1.26772600

C 3.50767200 -0.60085400 -1.10917700

C 4.76037400 0.59652000 1.10038600

C 4.78102900 -0.02943400 -1.22205400

C 5.40866100 0.56358800 -0.13049400

H 5.25005900 1.06855900 1.94802800

H 5.28688200 -0.04304200 -2.18375000

H 6.39651200 1.00291900 -0.24022800

C 2.80166600 0.13604300 2.63061100

C 2.43479400 1.59604900 2.96922800

C 3.64944500 -0.48628700 3.75710800

H 1.86860600 -0.43411600 2.57819800

H 1.80798000 2.04894500 2.19242500

H 1.89049000 1.64541800 3.92023400

H 3.33340800 2.21688800 3.06617300

H 3.90340100 -1.53038900 3.54108200

H 4.58815300 0.05953000 3.90661200

H 3.09925100 -0.46115900 4.70522900

C 2.84429100 -1.20446700 -2.34597800

C 3.73101200 -2.25668700 -3.03927900

C 2.42704400 -0.10294900 -3.34283900

H 1.93261500 -1.71601500 -2.02163700

H 4.03403600 -3.04940500 -2.34563200

H 3.18764800 -2.72313000 -3.86958000

H 4.64305000 -1.81178400 -3.45363400

H 1.76590500 0.63760000 -2.87797600

H 3.30278600 0.43651600 -3.72244600

H 1.90233600 -0.53815900 -4.20211300

C -2.85980500 -0.56716400 0.15246400

C -3.48383600 0.04204900 1.26883700

C -3.50772600 -0.60329400 -1.10717000

C -4.75873400 0.59729800 1.10157300

C -4.78119700 -0.03204000 -1.21992100

C -5.40797900 0.56262400 -0.12874300

H -5.24762600 1.07087200 1.94880900

H -5.28778900 -0.04699500 -2.18124100

H -6.39584000 1.00196200 -0.23841300

C -2.79907700 0.13857400 2.63105900

C -3.64638200 -0.48163900 3.75914000

C -2.43135600 1.59889500 2.96731600

H -1.86628000 -0.43202900 2.57882000

H -3.90156700 -1.52575900 3.54470900

H -3.09526600 -0.45582200 4.70670700

H -4.58439800 0.06529600 3.90884500

H -1.80468700 2.05028100 2.18954600

H -3.32962700 2.22028700 3.06383800

H -1.88655300 1.64935400 3.91798000

C -2.84537400 -1.20846000 -2.34378200

C -2.43177000 -0.10846300 -3.34381600

C -3.73167800 -2.26368800 -3.03306500

H -1.93228800 -1.71776500 -2.01992700

H -1.77144500 0.63442600 -2.88156500

H -1.90743700 -0.54464200 -4.20282400

H -3.30915200 0.42830400 -3.72347400

H -4.03167500 -3.05559400 -2.33718400

H -4.64551300 -1.82126600 -3.44611500

H -3.18937500 -2.73081400 -3.86367800

C -1.17427100 2.88840500 -0.77029600

C -1.23410200 4.19102900 -1.10412900

H -2.09748100 2.32662900 -0.63096300

C 1.22901400 4.19390200 -1.10005200

C -0.00296300 5.04311300 -1.31589000

H -2.21242000 4.65047500 -1.22673800

H 2.20683900 4.65542500 -1.21888500

H -0.00203600 5.49089100 -2.32853400

N -0.00108200 2.14812700 -0.57636300

C 1.17100700 2.89102300 -0.76653700

H 2.09505400 2.33128700 -0.62441300

H -0.00511600 5.91688700 -0.63560500

**23-ts**

C -1.40186100 -1.77735400 -2.00977600

C -0.13333400 -2.04456100 -2.57065100

C 1.17310900 -1.83929200 -2.07653300

H -0.16925400 -2.67753600 -3.45100900

N -1.62988400 -0.96670100 -0.96812400

C -2.54596000 -2.51030200 -2.69957200

H -2.21192700 -3.47767600 -3.08295500

H -3.39792800 -2.66883200 -2.03755400

H -2.89406200 -1.92120600 -3.55611500

C 2.22844600 -2.68348900 -2.78210200

H 2.08777400 -2.62305100 -3.86613500

H 3.24758400 -2.38355100 -2.54110100

H 2.10895400 -3.73681700 -2.50399400

N 1.49678700 -1.03862300 -1.05342400

C -2.92171400 -0.99732500 -0.31796600

C -3.07141700 -1.81536100 0.83685600

C -4.01801500 -0.22226200 -0.77768200

C -4.30233800 -1.83022100 1.50317900

C -5.22653100 -0.27436900 -0.06771500

C -5.37718400 -1.06457200 1.06295000

H -4.42612900 -2.45384700 2.38232900

H -6.06798200 0.31710000 -0.41781600

H -6.32419500 -1.08934100 1.59567600

C -1.94364100 -2.71775400 1.33886400

C -1.93116600 -4.07386700 0.60016500

C -1.98841400 -2.96947200 2.85692900

H -0.99452800 -2.21792800 1.11384000

H -1.76312400 -3.95547900 -0.47324700

H -1.13423800 -4.71772600 0.99327900

H -2.88515600 -4.59705000 0.73967000

H -2.10080400 -2.03955200 3.42283300

H -2.81325800 -3.63543200 3.13625200

H -1.06273000 -3.45676800 3.18165500

C -3.97792400 0.65158700 -2.03059900

C -4.29077300 2.12975700 -1.71550400

C -4.96277800 0.15333200 -3.11173000

H -2.96795600 0.59460600 -2.44932000

H -3.62787400 2.53776000 -0.94875500

H -4.18064200 2.74270900 -2.61822400

H -5.32273300 2.24535100 -1.36421300

H -4.81009400 -0.90050100 -3.36041700

H -6.00123700 0.26507600 -2.77823700

H -4.84691900 0.74145500 -4.03014800

C 2.83076300 -1.09776700 -0.51040600

C 3.79673800 -0.12263600 -0.88124600

C 3.16500500 -2.11349500 0.42380800

C 5.06853400 -0.18774900 -0.29645900

C 4.45101700 -2.12347300 0.98120400

C 5.39985800 -1.17172500 0.63017100

H 5.81764200 0.54664000 -0.57336000

H 4.71285800 -2.89828300 1.69731200

H 6.39361300 -1.19767200 1.06958500

C 3.50819900 0.96117500 -1.92226400

C 3.78861400 0.47730100 -3.36175900

C 4.29023100 2.26450600 -1.67527100

H 2.44263500 1.20497600 -1.86803900

H 3.14251100 -0.35395400 -3.65284300

H 3.61642400 1.29549000 -4.07109900

H 4.83113400 0.15205200 -3.46786500

H 4.22662900 2.59146000 -0.63215800

H 5.35154400 2.15795600 -1.93149000

H 3.87755200 3.06263900 -2.29906400

C 2.19547100 -3.22304300 0.83229400

C 1.90146300 -3.20374000 2.34448500

C 2.71900900 -4.61640200 0.42431000

H 1.24870300 -3.05897100 0.31050100

H 1.45564000 -2.25316800 2.65477400

H 1.20329900 -4.00645300 2.60952000

H 2.81330100 -3.35168500 2.93454500

H 2.95168300 -4.66681800 -0.64367800

H 3.63260200 -4.87519600 0.97264900

H 1.97004800 -5.38622900 0.64639100

H -2.11060600 1.82932900 0.58500900

B -1.12176500 2.03860500 1.20764100

O -1.03453100 3.06987800 2.09633300

O -0.24915100 0.96206700 1.57908400

C -0.05423300 2.80255500 3.12380400

C 0.36861900 1.27776000 2.87892700

C 1.08161900 3.82337700 2.97148500

H 0.66296000 4.82618700 3.10044500

H 1.85790000 3.67846000 3.73074600

H 1.53403400 3.78894100 1.98039100

C 1.87628600 1.04538000 2.78325800

H 2.35299200 1.24339800 3.74956700

H 2.08432300 0.00439400 2.51852300

H 2.35062200 1.68437100 2.03642300

C -0.23856200 0.29490400 3.88495300

H 0.03131500 -0.72662400 3.60318000

H 0.13800800 0.47745400 4.89645900

H -1.32956300 0.36046200 3.90112800

C -0.75170600 3.04742700 4.46920000

H -0.07358200 2.86717300 5.31083700

H -1.07392300 4.09207700 4.51099300

H -1.63827400 2.42135200 4.59040300

Mg -0.01193100 0.25154200 -0.39913400

C 1.12365200 3.08642400 -0.53647200

C 1.37955200 4.30022000 -1.05937500

H 1.84519500 2.63960100 0.14432800

C -0.66923100 4.02940400 -2.38745800

C 0.44089200 4.98638400 -2.02344000

H 2.29997400 4.80634300 -0.77745900

H -1.37686800 4.32286900 -3.15963000

H 0.98225100 5.32297900 -2.92635000

N -0.00287000 2.29237700 -0.78828400

C -0.82630700 2.83505100 -1.79085100

H -1.65020700 2.19437500 -2.08612700

H 0.03339000 5.91795600 -1.58601900

**24**

C 1.62205400 1.21134400 -2.05414000

C 0.38927200 1.32933300 -2.74378600

C -0.94764400 1.28998900 -2.30050900

H 0.49747900 1.73130500 -3.74675700

Mg 0.07032600 -0.37289800 -0.16622400

N 1.78643000 0.61607800 -0.86969100

C 2.77795100 1.88429000 -2.78557700

H 2.53024700 2.93151400 -2.98845600

H 3.71304100 1.85115400 -2.22959300

H 2.92989400 1.39977600 -3.75673000

C -1.93035500 1.89361300 -3.29992600

H -1.94858400 1.27287500 -4.20369600

H -2.94434600 1.96477000 -2.90997400

H -1.60388900 2.89205700 -3.60657800

N -1.36661600 0.80304300 -1.11892800

C 3.05753500 0.72799300 -0.18388500

C 3.19772000 1.71153000 0.83255000

C 4.15352500 -0.12166200 -0.49280000

C 4.42378400 1.83706600 1.49634400

C 5.35942800 0.05420500 0.20145300

C 5.50541700 1.02125900 1.18610500

H 4.53737900 2.59449600 2.26519900

H 6.20305200 -0.58555600 -0.04115800

H 6.45117900 1.13803800 1.70852400

C 2.07730300 2.68828100 1.18101700

C 2.21155400 3.98904600 0.36123900

C 2.00399700 3.01739300 2.68380500

H 1.12692900 2.22255800 0.89955100

H 2.16250100 3.79468000 -0.71435000

H 1.41088700 4.69452800 0.61374700

H 3.17028900 4.47907400 0.57017800

H 1.99656100 2.10964700 3.29629300

H 2.84834800 3.63440300 3.01176300

H 1.09222000 3.58325600 2.90081900

C 4.11310200 -1.22550300 -1.55002500

C 4.55142700 -2.58457100 -0.95887700

C 5.00292300 -0.90952000 -2.77140700

H 3.08291500 -1.32350100 -1.90320200

H 4.04509500 -2.80856300 -0.01468900

H 4.33917000 -3.39534700 -1.66606400

H 5.62944600 -2.60267600 -0.76135500

H 4.71170700 0.01744200 -3.27109100

H 6.05435400 -0.81181400 -2.47578400

H 4.93902800 -1.72231300 -3.50508000

C -2.68437900 1.17167400 -0.64456600

C -3.80632500 0.31871100 -0.82390200

C -2.85004700 2.41854500 0.02033400

C -5.05274600 0.72965800 -0.32998400

C -4.11963000 2.77585600 0.49219500

C -5.21878600 1.94296600 0.32549900

H -5.91498900 0.08592100 -0.47423600

H -4.24999700 3.73155000 0.99183700

H -6.19657500 2.23961300 0.69578000

C -3.74178800 -1.00240400 -1.58709600

C -4.30607200 -0.86412200 -3.01772900

C -4.48864600 -2.13927900 -0.86266300

H -2.69283200 -1.29756900 -1.67309200

H -3.76787400 -0.11513700 -3.60402900

H -4.23374400 -1.82175500 -3.54746500

H -5.36340400 -0.57266400 -2.99367500

H -4.17049800 -2.24084200 0.17918700

H -5.57304800 -1.98016900 -0.86470100

H -4.30329000 -3.09116200 -1.37071000

C -1.71367100 3.42371800 0.20109700

C -1.59738500 3.92204500 1.65438500

C -1.87443100 4.62829300 -0.75051000

H -0.77579200 2.92519100 -0.05749200

H -1.48131400 3.09145700 2.35758200

H -0.73069300 4.58426700 1.75949700

H -2.47889500 4.49647200 1.96050000

H -1.92325300 4.31542900 -1.79755100

H -2.79305500 5.18444700 -0.52747100

H -1.02961900 5.31963500 -0.64312000

H 1.54534000 -2.81086400 1.49797100

B 0.37022000 -2.53257600 1.35726500

O -0.54160600 -3.21534500 2.23431800

O 0.11044400 -1.04377900 1.68576400

C -1.13029500 -2.33895200 3.19555700

C -0.28422500 -1.00603300 3.07696700

C -2.61121400 -2.13226100 2.83051200

H -3.08309100 -3.11479300 2.72580800

H -3.15444300 -1.57300800 3.60086400

H -2.72354400 -1.59908500 1.88146600

C -1.06146100 0.27957600 3.35217300

H -1.45008300 0.28143100 4.37703300

H -0.39881200 1.14540400 3.24818800

H -1.90405200 0.40616700 2.66726000

C 0.99674300 -1.03133900 3.92534900

H 1.63591600 -0.19128400 3.63507300

H 0.76796500 -0.93905900 4.99214500

H 1.56272800 -1.95226900 3.76702500

C -1.04890900 -3.00470700 4.57556700

H -1.43315800 -2.34771600 5.36565900

H -1.65429000 -3.91729700 4.57112900

H -0.02302000 -3.28864100 4.82072200

C 1.10808100 -3.07339300 -1.04942000

C -1.43343500 -3.86718900 -1.72614900

C 0.95513300 -3.66510100 -2.23999900

H 2.08405500 -2.90016800 -0.62274600

C -0.40106100 -3.97788800 -2.82163000

H -2.40396200 -4.33100900 -1.87933100

H 1.84506500 -3.96114600 -2.78948100

H -0.40749200 -4.99121100 -3.25275700

N 0.04121400 -2.64504500 -0.20784500

C -1.20230500 -3.24344300 -0.56376500

H -1.94607800 -3.20452500 0.22069400

H -0.64082100 -3.30940400 -3.66895600

**25-ts**

C -1.95648000 -2.51655900 -0.82029900

C -0.84919900 -3.37281600 -0.66257700

C 0.47578600 -3.12251100 -0.25456900

H -1.04857600 -4.40826500 -0.91372100

N -1.97673000 -1.21328200 -0.50939600

C -3.17328400 -3.18520300 -1.45012300

H -3.20769800 -4.24899900 -1.20119200

H -4.11687000 -2.72269500 -1.16104300

H -3.09251800 -3.10826000 -2.54167300

C 1.35926700 -4.36119500 -0.18083300

H 0.75170200 -5.25487200 -0.01831600

H 1.90539800 -4.49844000 -1.12119000

H 2.10380900 -4.29079100 0.61441700

N 0.98000700 -1.91236200 0.01231400

C -3.23479500 -0.51653000 -0.57838700

C -4.07687900 -0.50215200 0.57015500

C -3.61712300 0.17600900 -1.75533700

C -5.28180600 0.20835600 0.50733000

C -4.83261200 0.87514500 -1.75707900

C -5.66243200 0.89554500 -0.64269600

H -5.93776300 0.22525400 1.37239600

H -5.13453900 1.40729900 -2.65524200

H -6.60267700 1.44030900 -0.66811900

C -3.71651100 -1.25857700 1.85372400

C -4.25762600 -2.70534800 1.85986100

C -4.19981300 -0.55192600 3.13601800

H -2.62362400 -1.32665600 1.89617000

H -3.81172200 -3.31653300 1.07311000

H -4.03508400 -3.18776300 2.81966800

H -5.34616800 -2.71362200 1.72412900

H -3.95083400 0.51338600 3.14520300

H -5.28528200 -0.64048000 3.26277100

H -3.73745000 -1.01835900 4.01367400

C -2.78162100 0.16825200 -3.03581100

C -2.34499400 1.58478700 -3.45750100

C -3.53326400 -0.51919200 -4.19542600

H -1.87085000 -0.40603800 -2.84202800

H -1.73203600 2.05716900 -2.68569300

H -1.74314700 1.53425600 -4.37277400

H -3.21020200 2.22677700 -3.66387900

H -3.86299800 -1.52913200 -3.93000800

H -4.42327500 0.05124300 -4.48759500

H -2.88459600 -0.59321400 -5.07640700

C 2.37872000 -1.80776900 0.32402200

C 3.34642700 -1.83377200 -0.71280700

C 2.77384700 -1.63101500 1.67588900

C 4.69709300 -1.69814300 -0.36639300

C 4.13837700 -1.50795100 1.96569600

C 5.09773400 -1.54305100 0.95655000

H 5.44870800 -1.71318800 -1.15081900

H 4.45901000 -1.38356200 2.99535400

H 6.15225100 -1.44742800 1.20212600

C 2.97353400 -1.99310700 -2.18827900

C 3.78702800 -3.10436600 -2.88310500

C 3.11967300 -0.66762300 -2.96329300

H 1.91955100 -2.27977600 -2.24238300

H 3.74220300 -4.05252300 -2.33546400

H 3.39977900 -3.27666700 -3.89422900

H 4.84405000 -2.83151000 -2.98335600

H 2.47762700 0.11475900 -2.54901600

H 4.15390000 -0.30474700 -2.93443400

H 2.84240300 -0.81497500 -4.01478500

C 1.74410600 -1.61035200 2.80568500

C 2.14676000 -0.70101400 3.98009500

C 1.43534700 -3.02766800 3.33194300

H 0.81204900 -1.21461300 2.38520400

H 2.45569700 0.29138200 3.63582000

H 1.30163400 -0.58068500 4.66846300

H 2.97300000 -1.12533500 4.56245800

H 1.01982300 -3.67016900 2.55124600

H 2.34500000 -3.50474600 3.71691900

H 0.70612700 -2.98291400 4.15050600

H 0.22419800 1.06644300 -1.55481200

B 0.83816600 2.52855500 -0.05138200

O -0.04959500 3.58478300 -0.08827900

O 0.45196500 1.56714700 0.94791800

C -1.00548600 3.44593700 0.98748400

C -0.31930400 2.37089800 1.92176300

C -1.17428900 4.82079400 1.64113900

H -1.63142700 5.50752000 0.92193900

H -1.83165600 4.76253100 2.51660300

H -0.21593000 5.24578500 1.94728100

C 0.69743400 2.97841700 2.89669600

H 0.19730500 3.56607700 3.67316900

H 1.25419200 2.17321800 3.38341700

H 1.41450700 3.62484800 2.38211200

C -1.27888500 1.45435300 2.66163400

H -0.72385100 0.71348200 3.24692400

H -1.89676600 2.03369300 3.35667000

H -1.94603100 0.93094600 1.97531600

C -2.34080000 2.99971300 0.38647300

H -3.13314700 2.95053800 1.14050900

H -2.63857700 3.72713800 -0.37471900

H -2.27243000 2.02724500 -0.10421200

Mg -0.13241300 -0.17580700 -0.35742000

C 3.25359900 1.87599200 -0.18486900

C 4.50608400 2.06498600 -0.62055300

H 3.00361300 1.06405100 0.48730800

C 3.69529200 4.03011300 -1.88152400

C 4.90131700 3.18432200 -1.55293700

H 5.27001300 1.37049300 -0.28493100

H 3.82327700 4.88270400 -2.54317400

H 5.35655300 2.78004000 -2.47487600

C 2.47288900 3.78456400 -1.39511000

H 1.61973500 4.40620300 -1.63874300

N 2.17859800 2.71402100 -0.52681200

H 5.69796800 3.80294300 -1.10182000

**26**

B 0.10383300 0.00000700 0.00036700

O -0.64915900 -1.13138200 0.22692100

O -0.64905800 1.13133900 -0.22678500

C -2.02645400 -0.78564800 -0.08809000

C -2.02647300 0.78562200 0.08780300

C -2.27979100 -1.23293700 -1.53422400

H -2.06083300 -2.30200600 -1.61814500

H -3.32082800 -1.07002400 -1.83276800

H -1.63020100 -0.69926200 -2.23539700

C -2.94397500 1.54605500 -0.86782500

H -3.99191500 1.26767300 -0.70708800

H -2.84891500 2.62218500 -0.68977800

H -2.68744600 1.35392200 -1.91208900

C -2.28010700 1.23279800 1.53391100

H -2.06102300 2.30183300 1.61801500

H -3.32125200 1.06999100 1.83214100

H -1.63079500 0.69894200 2.23522400

C -2.94416800 -1.54596000 0.86741100

H -3.99208700 -1.26774600 0.70625300

H -2.84889700 -2.62211900 0.68964700

H -2.68801800 -1.35354400 1.91173000

C 2.27187400 -1.18757300 0.16670600

C 3.60928200 -1.22651700 0.17167800

H 1.66313900 -2.07590200 0.29099600

C 3.60914600 1.22647100 -0.17215800

C 4.47401500 0.00007200 -0.00066500

H 4.09603600 -2.18877200 0.30547600

H 4.09572900 2.18870300 -0.30684600

H 5.15015900 -0.12141400 -0.86542000

C 2.27175200 1.18763600 -0.16563700

H 1.66284600 2.07592300 -0.28925300

N 1.53131100 0.00004900 0.00108100

H 5.15109200 0.12145600 0.86336100

**27-ts**

C -3.69739300 1.36332400 0.32658000

C -3.10099200 2.61662800 0.59028700

C -1.75118900 3.01829300 0.59763500

H -3.81163100 3.41646900 0.76752900

Mg -0.98184800 0.19224700 0.22932600

N -3.01713200 0.23105800 0.12318900

C -5.21840200 1.38282900 0.28748500

H -5.64419500 0.42158500 0.00077800

H -5.61220800 1.65513000 1.27385600

H -5.56584400 2.14559100 -0.41777400

C -1.50675900 4.50298900 0.81211600

H -2.39683200 4.98890000 1.21777900

H -0.66665500 4.67741000 1.48951200

H -1.25600300 4.99512500 -0.13473500

N -0.70881100 2.19560200 0.40185700

C -3.69947100 -0.99834400 -0.19264200

C -4.09050200 -1.87796900 0.84593200

C -3.90186000 -1.35009000 -1.54967000

C -4.70734100 -3.08686000 0.50047000

C -4.51980600 -2.57257100 -1.84009800

C -4.92669500 -3.43646200 -0.82817700

H -5.01790100 -3.76754700 1.28874700

H -4.68383100 -2.85234700 -2.87750600

H -5.40652300 -4.37999900 -1.07385400

C -3.86469800 -1.55516300 2.32119500

C -3.00489700 -2.62952300 3.01709200

C -5.19652000 -1.35653800 3.07332600

H -3.31848900 -0.60744400 2.37710200

H -2.05315200 -2.78729900 2.49767900

H 2.78930700 -2.33223700 4.05090200

H -3.52478300 -3.59400400 3.05556700

H -5.80628900 -0.57053500 2.61464600

H -5.79129700 -2.27781200 3.07794600

H -5.00937100 -1.07655600 4.11699500

C -3.46008500 -0.44849000 -2.70049200

C -4.64725900 -0.00543200 -3.57774400

C -2.36850600 -1.12326500 -3.55549000

H -3.02520500 0.45827200 -2.26845100

H -5.41330700 0.50554400 -2.98401100

H -4.30957200 0.68296800 -4.36176800

H -5.12416200 -0.86080300 -4.07037100

H 1.51462600 -1.43544800 -2.94238800

H -2.75134500 -2.01921500 -4.05785200

H -2.00394800 -0.43703600 -4.32965500

C 0.61980500 2.74256100 0.31566600

C 1.49681200 2.63099800 1.42262600

C 1.06168700 3.32700600 -0.89869400

C 2.78180400 3.17872800 1.31389000

C 2.36027800 3.84560900 -0.95851300

C 3.21235600 3.79131000 0.14108200

H 3.45831600 3.11706700 2.16108500

H 2.71260700 4.29770000 -1.88140100

H 4.21301400 4.21062800 0.07870200

C 1.08336700 1.94540700 2.72334700

C 0.67654600 2.96868500 3.80359200

C 2.16681200 0.99101800 3.26199300

H 0.19263900 1.34166600 2.50893400

H -0.16184800 3.59384000 3.47810000

H 0.37625700 2.45618100 4.72560500

H 1.51533700 3.63222800 4.04664600

H 2.52708400 0.29971400 2.49359000

H 3.03136300 1.54237300 3.65072300

H 1.76325200 0.40322400 4.09527500

C 0.18716500 3.34752500 -2.15247600

C 0.61621100 2.22959200 -3.12615400

C 0.18151900 4.70998800 -2.87097300

H -0.84403800 3.13785900 -1.85379700

H 0.57790600 1.24256600 -2.64893600

H -0.03736900 2.20577700 -4.00691000

H 1.64533500 2.38278800 -3.47146200

H -0.09304300 5.52426300 -2.19088200

H 1.16049300 4.95250600 -3.29981000

H -0.54077000 4.69917200 -3.69565900

C 0.45426400 -2.08005500 1.44974900

C 1.05371600 -3.33490800 1.37260200

H 0.19339200 -1.62989100 2.40851600

C 0.73660100 -3.31843100 -1.01161300

C 0.76487400 -4.05479800 0.11480400

H 1.12376400 -3.91338500 2.29198500

H 0.47727300 -3.70570200 -1.99315200

H 0.58412100 -5.12619500 0.13859900

N 0.31566500 -1.29518000 0.32745800

C 1.11233900 -1.88647500 -0.76968900

H 1.13739600 -1.23992500 -1.64924000

B 3.53262500 -2.52961200 0.42509200

H 2.32488100 -3.11504200 1.11657700

O 4.09361300 -1.32943000 0.86727200

O 4.26650100 -3.06419700 -0.63012600

C 5.10092900 -0.91270200 -0.09850300

C 5.47521900 -2.27492500 -0.79568400

C 4.44504400 0.09865800 -1.04971500

H 4.03920300 0.93044900 -0.46629000

H 5.16570000 0.50276900 -1.77003000

H 3.62123600 -0.35636900 -1.60745600

C 5.79266400 -2.18004700 -2.28864000

H 6.67690400 -1.55615600 -2.46830800

H 5.99978900 -3.18100300 -2.68188100

H 4.95417400 -1.76632800 -2.85398300

C 6.59565200 -3.04011100 -0.07380200

H 6.65066000 -4.05339000 -0.48428300

H 7.57242300 -2.56037700 -0.20554500

H 6.38756700 -3.12302300 0.99732500

C 6.23791000 -0.23590300 0.66770700

H 7.05817300 0.04205100 -0.00532700

H 5.86537300 0.67840000 1.14204700

H 6.63507400 -0.88344000 1.45278700

H 2.33574600 -1.95446100 -0.32733400

**6a**

C 1.28849200 -0.00063500 1.81508300

C -0.00000500 -0.00078600 2.39611900

C -1.28850200 -0.00072400 1.81506100

H -0.00000500 -0.00193100 3.47961300

N 1.53486600 0.00095800 0.50341100

C 2.45597000 -0.00297400 2.79106200

H 3.09077400 -0.88266600 2.63666800

H 3.09630100 0.87229500 2.63466400

H 2.11349400 -0.00085900 3.82792100

C -2.45596300 -0.00313200 2.79106400

H -2.11347100 -0.00138400 3.82791700

H -3.09612000 0.87231300 2.63495100

H -3.09095000 -0.88264000 2.63642500

N -1.53488300 0.00088400 0.50342500

C 2.88090600 -0.00001400 0.01453700

C 3.52216500 1.22923400 -0.29215600

C 3.52035700 -1.23009400 -0.29257900

C 4.79053900 1.20083300 -0.88419300

C 4.78875500 -1.20333700 -0.88462300

C 5.42699600 -0.00166300 -1.17781400

H 5.28721600 2.13758800 -1.12398300

H 5.28404800 -2.14072900 -1.12478400

H 6.41119300 -0.00230900 -1.63840300

C 2.84911800 2.57398000 -0.02226400

C 2.44744000 3.26968900 -1.33984800

C 3.72009500 3.51114000 0.83611200

H 1.93145300 2.37872500 0.54174900

H 1.79902000 2.63629900 -1.96062700

H 1.91364400 4.20677500 -1.14004200

H 3.32850300 3.50646100 -1.94750800

H 4.00969400 3.03521200 1.77954400

H 4.63921700 3.80330100 0.31495000

H 3.17106100 4.42983600 1.07492800

C 2.84513400 -2.57388300 -0.02346600

C 3.71529800 -3.51379700 0.83272500

C 2.44064900 -3.26714200 -1.34145700

H 1.92842700 -2.37765000 0.54170600

H 4.00722200 -3.03949900 1.77626500

H 3.16463100 -4.43156800 1.07132300

H 4.63308900 -3.80742300 0.31004500

H 1.79255700 -2.63170600 -1.96046800

H 3.32053300 -3.50462800 -1.95054800

H 1.90536800 -4.20351000 -1.14228900

C -2.88090500 -0.00011200 0.01455200

C -3.52225800 1.22912500 -0.29194800

C -3.52025000 -1.23018100 -0.29277200

C -4.79061800 1.20072700 -0.88401400

C -4.78864200 -1.20344300 -0.88483300

C -5.42698200 -0.00177400 -1.17784500

H -5.28736600 2.13748200 -1.12366700

H -5.28385600 -2.14083600 -1.12515400

H -6.41117200 -0.00242300 -1.63845500

C -2.84931800 2.57388100 -0.02185500

C -3.72043500 3.51092200 0.83650800

C -2.44754900 3.26973300 -1.33933800

H -1.93169700 2.37864200 0.54222500

H -4.01015300 3.03488400 1.77984600

H -3.17145500 4.42960500 1.07550100

H -4.63949200 3.80312600 0.31525600

H -1.79900700 2.63645000 -1.96009600

H -3.32856500 3.50648100 -1.94707700

H -1.91385100 4.20684600 -1.13939800

C -2.84494300 -2.57396300 -0.02385300

C -2.44053800 -3.26708800 -1.34194000

C -3.71500300 -3.51399800 0.83231600

H -1.92820300 -2.37775400 0.54126600

H -1.79253300 -2.63157300 -1.96095900

H -1.90519400 -4.20344700 -1.14289700

H -3.32046200 -3.50457300 -1.95097500

H -4.00683900 -3.03982400 1.77594400

H -4.63284200 -3.80757200 0.30969100

H -3.16429800 -4.43179000 1.07074400

H 0.00003800 0.01313600 -3.45675500

Ca 0.00000700 0.00633900 -1.34314800

**13a**

C -1.29566700 -1.08174000 1.88660900

C 0.00000800 -1.20317400 2.44306900

C 1.29567000 -1.08194100 1.88655800

H 0.00005100 -1.58265900 3.45837200

N -1.57998700 -0.60636800 0.67270200

C -2.41816300 -1.60914400 2.77318800

H -3.30398500 -0.97073200 2.73475200

H -2.73333400 -2.60295000 2.43175800

H -2.09275300 -1.70142900 3.81196900

C 2.41822700 -1.60956800 2.77296600

H 2.09300700 -1.70161300 3.81183200

H 2.73295600 -2.60353900 2.43154500

H 3.30425600 -0.97144100 2.73421300

N 1.57997200 -0.60652600 0.67254200

C -2.92347700 -0.67798100 0.17850700

C -3.34378100 -1.82326300 -0.55012900

C -3.80601200 0.42570000 0.32421200

C -4.63641000 -1.84930900 -1.08699500

C -5.09170200 0.34466500 -0.22535200

C -5.51391400 -0.78196900 -0.92364500

H -4.96096900 -2.72420500 -1.64459200

H -5.77485500 1.18180300 -0.10426800

H -6.51564200 -0.82489700 -1.34303200

C -2.42386900 -3.02054400 -0.78520600

C -2.08463900 -3.18262500 -2.28168000

C -3.01399600 -4.32866400 -0.22216700

H -1.48638800 -2.83105900 -0.25275500

H -1.62363500 -2.27845200 -2.69637500

H -1.38699800 -4.01642400 -2.42853400

H -2.98449500 -3.39397900 -2.87201800

H -3.24603600 -4.24153000 0.84515100

H -3.93882200 -4.60853500 -0.74037200

H -2.30250300 -5.15371400 -0.34765800

C -3.39879600 1.70159900 1.05909900

C -4.21217800 1.91945400 2.35141800

C -3.51573600 2.94109400 0.14925200

H -2.34768200 1.59284700 1.34781200

H -4.10317400 1.08068000 3.04654300

H -3.87904900 2.82863100 2.86742600

H -5.28062300 2.03369600 2.13288100

H -2.94103700 2.81761000 -0.77489600

H -4.55716200 3.13343500 -0.13309100

H -3.14441000 3.83532600 0.66486300

C 2.92348500 -0.67810300 0.17825200

C 3.34358800 -1.82315200 -0.55092800

C 3.80621400 0.42541600 0.32445500

C 4.63616300 -1.84908000 -1.08793000

C 5.09185200 0.34441100 -0.22525900

C 5.51380100 -0.78194400 -0.92414500

H 4.96053300 -2.72374700 -1.64598100

H 5.77514000 1.18138200 -0.10380100

H 6.51546900 -0.82479600 -1.34365400

C 2.42350800 -3.02024600 -0.78633000

C 3.01327700 -4.32846100 -0.22312800

C 2.08458400 -3.18221900 -2.28287000

H 1.48593600 -2.83057600 -0.25406700

H 3.24504300 -4.24131900 0.84425500

H 2.30168500 -5.15340200 -0.34879000

H 3.93819900 -4.60846500 -0.74108100

H 1.62383000 -2.27794200 -2.69763300

H 2.98452400 -3.39372300 -2.87303000

H 1.38682000 -4.01588100 -2.42989600

C 3.39919700 1.70105900 1.05993600

C 3.51602900 2.94087600 0.15052400

C 4.21278400 1.91835300 2.35221100

H 2.34810400 1.59220600 1.34876900

H 2.94109500 2.81775400 -0.77353300

H 3.14488500 3.83495000 0.66654700

H 4.55740900 3.13322300 -0.13198100

H 4.10389600 1.07924900 3.04697300

H 5.28119800 2.03270900 2.13356200

H 3.87971500 2.82728700 2.86868700

H -0.00018000 0.17431300 -3.10282400

C 0.00044600 3.70561300 0.06084000

C 0.00033900 5.08696200 -0.11082000

C -0.00015000 5.59839400 -1.40965300

C -0.00049800 4.70869300 -2.48293000

C -0.00034100 3.33952000 -2.22003900

N 0.00011200 2.83988000 -0.96839000

H -0.00024800 6.67144100 -1.57996700

H 0.00084100 3.26880700 1.05636100

H 0.00064400 5.74184500 0.75460300

H -0.00087700 5.06059000 -3.50952700

H -0.00057400 2.59676700 -3.01751700

Ca 0.00002300 0.26197300 -0.96086500

**14a-ts**

C 0.99940200 -2.52227700 0.52958400

C -0.33084600 -2.99442900 0.56791800

C -1.56518700 -2.32837000 0.38126400

H -0.42076300 -4.06259900 0.72630900

N 1.35096000 -1.24788200 0.34543500

C 2.08376700 -3.57668800 0.69704600

H 2.67772000 -3.67522400 -0.21864400

H 2.78214600 -3.29516200 1.49258500

H 1.65937000 -4.55428700 0.93550400

C -2.80076500 -3.21650300 0.37783600

H -3.47532800 -2.95211600 1.19932900

H -3.37070800 -3.08324900 -0.54808600

H -2.53731800 -4.27185500 0.47443500

N -1.70350700 -1.01400700 0.20816400

C 2.72299600 -0.87124600 0.20319700

C 3.42698000 -0.33276900 1.31291900

C 3.33368900 -0.89517500 -1.08097600

C 4.71436100 0.18276400 1.11328800

C 4.62153100 -0.36643000 -1.22553600

C 5.31207000 0.17364500 -0.14300100

H 5.25746300 0.59711200 1.95907700

H 5.09359300 -0.37628400 -2.20483800

H 6.31070200 0.58046400 -0.27813100

C 2.82352400 -0.31206700 2.71673400

C 2.60689600 1.12520700 3.23292000

C 3.67522500 -1.11823400 3.71782600

H 1.84335200 -0.79822500 2.66285400

H 1.95219300 1.71063800 2.57391900

H 2.14788400 1.11200600 4.22888200

H 3.55574000 1.66912300 3.30835200

H 3.82312300 -2.14945900 3.37854100

H 4.66576600 -0.66902200 3.85568100

H 3.18562500 -1.15038300 4.69849600

C 2.61601300 -1.46667300 -2.30401900

C 3.44605200 -2.55377700 -3.01524400

C 2.22151900 -0.36026400 -3.30353600

H 1.69078800 -1.93678100 -1.95856700

H 3.73834900 -3.35363300 -2.32586700

H 2.86479200 -3.00336400 -3.82904000

H 4.36316900 -2.14364100 -3.45429100

H 1.55958100 0.38667800 -2.84906200

H 3.10513800 0.16875500 -3.68050300

H 1.69535500 -0.78796800 -4.16575200

C -2.99307600 -0.42903500 -0.00843600

C -3.76994600 0.02142900 1.09321300

C -3.42983600 -0.17242700 -1.33630300

C -4.95903200 0.71622700 0.84065600

C -4.62677800 0.52787200 -1.53214400

C -5.39070100 0.97333500 -0.45763800

H -5.55732900 1.06566400 1.67848700

H -4.96626700 0.72490300 -2.54601500

H -6.31635600 1.51601900 -0.63023100

C -3.34173900 -0.21778700 2.54069100

C -4.40319300 -1.00099500 3.33891800

C -3.00140900 1.10402000 3.25980900

H -2.43182500 -0.82621600 2.52177600

H -4.65336700 -1.95280000 2.85731800

H -4.03628600 -1.21886700 4.34906800

H -5.33278200 -0.42888400 3.44178500

H -2.22050500 1.66601200 2.73270400

H -3.88227200 1.75327600 3.33265200

H -2.64933900 0.90574500 4.27991100

C -2.63672000 -0.64302100 -2.55577300

C -2.12325200 0.54049400 -3.40191600

C -3.45501700 -1.61356300 -3.43089700

H -1.76366900 -1.19423600 -2.19194600

H -1.48227200 1.21791600 -2.82419400

H -1.53839500 0.17556700 -4.25514800

H -2.95390900 1.13559600 -3.79949700

H -3.80204900 -2.47802100 -2.85411400

H -4.33819600 -1.12369400 -3.85768200

H -2.84589100 -1.98461100 -4.26396700

H -0.48152000 2.67247300 1.12899600

C 1.67814500 3.07585300 -1.23811600

C 2.18518300 4.26623300 -0.74726700

C 1.26785500 5.18982900 -0.18238800

C -0.04178200 4.82717900 -0.01547900

C -0.43496300 3.47717100 -0.32633500

N 0.38268400 2.69469000 -1.14000500

H 1.60097900 6.18976300 0.08671200

H 2.32575500 2.39608300 -1.79064100

H 3.23124300 4.51650400 -0.88657600

H -0.77578000 5.50791100 0.40506300

H -1.49907500 3.30337000 -0.46995100

Ca -0.02976900 0.71061200 0.19831300

**15a**

C 1.21421200 -2.20635100 1.29696600

C -0.08540900 -2.69410700 1.56273200

C -1.36183600 -2.19749800 1.21053900

H -0.10746300 -3.62031500 2.12450800

N 1.48385700 -1.09269700 0.61214100

C 2.36359400 -3.04112600 1.84048600

H 3.01061600 -3.39150000 1.02849600

H 2.99681100 -2.44359100 2.50596400

H 2.00264300 -3.91041100 2.39391700

C -2.54781600 -3.05068000 1.63399800

H -2.22771200 -3.94718300 2.16883100

H -3.22812600 -2.48504600 2.28006900

H -3.13420100 -3.35858900 0.76057300

N -1.57940400 -1.06135200 0.54564400

C 2.83342500 -0.68761100 0.35833400

C 3.44716400 0.28008000 1.19646100

C 3.49994900 -1.13986900 -0.81235100

C 4.71791900 0.76064900 0.85571400

C 4.76704400 -0.62519300 -1.10930500

C 5.37942600 0.31544700 -0.28448600

H 5.19453500 1.50066800 1.49353700

H 5.28259000 -0.96291200 -2.00449700

H 6.36368500 0.70283400 -0.53307400

C 2.75065700 0.82151800 2.44355200

C 2.44079000 2.32799700 2.31257500

C 3.55883000 0.54803600 3.72703000

H 1.79761000 0.29107900 2.54571300

H 1.86974000 2.56633700 1.40415300

H 1.86563700 2.68153200 3.17696500

H 3.36188500 2.92011900 2.26187600

H 3.76051800 -0.52158300 3.85258500

H 4.52319900 1.06924800 3.71378100

H 3.00674800 0.89461900 4.60875700

C 2.84451700 -2.13625300 -1.76744400

C 3.75818200 -3.32774300 -2.11106300

C 2.36955600 -1.43482000 -3.05717400

H 1.95788900 -2.54084800 -1.27022100

H 4.10320200 -3.84046800 -1.20628100

H 3.21759200 -4.05564800 -2.72756200

H 4.64472000 -3.01551100 -2.67463400

H 1.68191500 -0.60393200 -2.84589200

H 3.21400100 -1.01102700 -3.61304600

H 1.84809200 -2.13834700 -3.71730300

C -2.90865300 -0.63945600 0.21953300

C -3.62806300 0.19882200 1.11196400

C -3.44409800 -0.93876300 -1.06078300

C -4.87647600 0.69294300 0.71545000

C -4.69717700 -0.41973500 -1.40842000

C -5.41657600 0.38561000 -0.53051900

H -5.43284100 1.33625500 1.39225900

H -5.11303100 -0.64618800 -2.38671400

H -6.38718200 0.77991300 -0.81877500

C -3.05043500 0.61097000 2.46483100

C -4.00479100 0.31555600 3.63742500

C -2.64775700 2.10100800 2.46103800

H -2.14156200 0.02433400 2.63127600

H -4.28834800 -0.74243800 3.66644700

H -3.52422000 0.56335500 4.59129600

H -4.92671600 0.90477100 3.57114300

H -1.95169100 2.34020500 1.64546400

H -3.52237500 2.74767700 2.32494300

H -2.16722100 2.37870900 3.40695500

C -2.66980900 -1.77428300 -2.07863100

C -2.22093000 -0.91349500 -3.27961300

C -3.46399300 -3.00282700 -2.56212100

H -1.76886400 -2.14988900 -1.58209500

H -1.64704600 -0.02814400 -2.97158100

H -1.59903000 -1.50006800 -3.96690900

H -3.08431700 -0.54279300 -3.84449000

H -3.76156200 -3.64131200 -1.72280400

H -4.37494600 -2.71171500 -3.09784300

H -2.85622300 -3.60560300 -3.24750200

C -1.10545500 3.40379800 -1.58626600

C -0.82729100 4.75266000 -1.58984400

H -2.13544900 3.06087800 -1.44922300

C 1.53120700 4.21632100 -1.49451400

C 0.54305900 5.13094100 -1.33565000

H -1.63126100 5.47950100 -1.53499100

H 2.56783900 4.40892600 -1.22947600

H 0.75348600 6.12193800 -0.93433700

N -0.15685400 2.42436800 -1.62554000

C 1.12474800 2.92763300 -2.16707600

H 1.00511600 3.08814300 -3.26197100

H 1.90043800 2.15220800 -2.05469200

Ca -0.01393000 0.50742500 -0.32297500

**16a-ts**

C -0.85937100 -3.18152900 -0.85362500

C 0.48555600 -3.60329900 -0.77800500

C 1.67143600 -2.95804200 -0.35511400

H 0.62681700 -4.65262400 -1.01574000

N -1.29540100 -1.93378700 -0.65544600

C -1.85064100 -4.30588100 -1.14204900

H -2.16226000 -4.78956700 -0.20813100

H -2.75454000 -3.94547700 -1.63659700

H -1.38772600 -5.07536800 -1.76614600

C 2.88915700 -3.87717800 -0.31791400

H 3.28778900 -4.01239200 -1.33039400

H 3.69252100 -3.47966700 0.30425200

H 2.61120900 -4.86779900 0.05378600

N 1.77526300 -1.67261500 -0.01651300

C -2.69957000 -1.67861300 -0.56810600

C -3.40476600 -1.98191500 0.62797900

C -3.36451100 -1.02253900 -1.64079100

C -4.75279700 -1.61724500 0.72823200

C -4.71676600 -0.69043200 -1.49052500

C -5.41059100 -0.97852400 -0.31822900

H -5.29861200 -1.83900000 1.64173100

H -5.23856100 -0.19387100 -2.30220500

H -6.45851700 -0.70673500 -0.22279800

C -2.72810800 -2.67073300 1.81448900

C -3.53914400 -3.86701700 2.34931300

C -2.43485100 -1.67318500 2.95183100

H -1.76269800 -3.05690400 1.47904300

H -3.77808700 -4.57886700 1.55170100

H -2.96846800 -4.39874300 3.12015000

H -4.48504600 -3.55063000 2.80414100

H -1.78950600 -0.85786300 2.60434600

H -3.35890500 -1.22313000 3.33449600

H -1.92929400 -2.17207500 3.78800500

C -2.64195400 -0.71254800 -2.95404300

C -3.19436400 0.51929700 -3.69204800

C -2.63639800 -1.92610800 -3.90762300

H -1.58945400 -0.50431300 -2.71400600

H -3.27658900 1.39011500 -3.03369300

H -2.53013100 0.78308100 -4.52253900

H -4.18404300 0.32459300 -4.12202500

H -2.11964800 -2.78473400 -3.47181800

H -3.66219000 -2.23090900 -4.14828500

H -2.13069800 -1.67106500 -4.84691700

C 3.02333600 -1.12444200 0.42091200

C 4.02701500 -0.74418200 -0.50961500

C 3.20190400 -0.85992900 1.80850600

C 5.17598000 -0.09963700 -0.03256400

C 4.37281100 -0.21866700 2.22992500

C 5.35499500 0.16600000 1.32025400

H 5.94443300 0.20050900 -0.73984700

H 4.52397500 -0.01751200 3.28587400

H 6.25494000 0.66715700 1.66665900

C 3.90860900 -1.01489000 -2.00970400

C 5.07082300 -1.88809000 -2.52739300

C 3.82733900 0.28942300 -2.82736900

H 2.97892900 -1.56648000 -2.18035600

H 5.17275200 -2.81660700 -1.95630200

H 4.90883000 -2.14992400 -3.57992600

H 6.02705900 -1.35521400 -2.46605000

H 2.99157800 0.91865200 -2.51010600

H 4.74277000 0.88347000 -2.72047500

H 3.70197900 0.06193300 -3.89332700

C 2.16410400 -1.31616500 2.83600800

C 2.12678000 -0.45250400 4.10862400

C 2.36557900 -2.79632300 3.22501700

H 1.17466000 -1.25468900 2.36085500

H 2.07633100 0.61704300 3.87908500

H 1.25184500 -0.71595900 4.71455100

H 3.01035500 -0.61652200 4.73626100

H 2.27395900 -3.45890000 2.36087200

H 3.35937200 -2.94484600 3.66456000

H 1.61784800 -3.10430800 3.96652700

H -1.94254400 1.85359800 -1.09575300

B -1.52758900 2.47721400 -0.16959300

O -1.87531000 3.76839100 0.06583800

O -1.05811800 1.82139100 0.99432700

C -1.42319800 4.14812200 1.39318600

C -1.26206000 2.75086500 2.12276900

C -0.11489400 4.93037800 1.23398200

H -0.30464300 5.79518400 0.59131800

H 0.25002000 5.29496100 2.20082400

H 0.66690200 4.33968600 0.75107800

C -0.05998600 2.65272600 3.05578700

H -0.14383300 3.38522500 3.86606300

H -0.01875600 1.65835100 3.51209000

H 0.87842200 2.83381500 2.52580000

C -2.53507300 2.28767300 2.83898500

H -2.40413800 1.26065000 3.18962400

H -2.75207100 2.91796900 3.70745500

H -3.39970700 2.30819400 2.16904300

C -2.49395700 5.05730600 2.00054600

H -2.23292000 5.34115000 3.02656700

H -2.56317100 5.97238800 1.40472500

H -3.47846000 4.58459400 2.00603000

C 1.73522500 2.72267400 -0.93309700

C 2.26888300 3.88510000 -1.42980100

H 2.16099000 2.29754000 -0.01562800

C 0.46378000 3.96396100 -3.04112800

C 1.48464800 4.59260600 -2.42141100

H 3.13144500 4.34373300 -0.95747500

H -0.21657900 4.48188700 -3.71275900

H 1.67499900 5.65178900 -2.58994500

N 0.63670500 2.07483100 -1.43479700

C 0.31576300 2.47470700 -2.82063100

H -0.70699100 2.14582200 -3.04938100

H 0.97842000 1.92593600 -3.52684300

Ca 0.07413500 0.04415000 -0.28957400

**17a**

C 1.91858600 1.85098100 -1.47639300

C 0.72325500 2.25921000 -2.14101000

C -0.65667500 2.19707300 -1.82527200

H 0.92728300 2.97165900 -2.93320900

N 2.03536500 0.84537600 -0.61176000

C 3.10785800 2.75161700 -1.80324400

H 3.01422300 3.69113100 -1.24447500

H 4.06804400 2.30679700 -1.54464800

H 3.10859300 3.01181600 -2.86623900

C -1.51104100 3.16194900 -2.64783700

H -1.85164700 2.66117900 -3.56259100

H -2.39922200 3.48802100 -2.10415100

H -0.94038900 4.04373000 -2.94868500

N -1.23209200 1.39566400 -0.91693500

C 3.27620300 0.68340700 0.10307100

C 3.46124900 1.34444700 1.34461700

C 4.30494900 -0.15583100 -0.40403400

C 4.67527600 1.19220000 2.02582600

C 5.50416000 -0.26315900 0.31255000

C 5.70098600 0.40623600 1.51527300

H 4.81792700 1.70621200 2.97275700

H 6.30166100 -0.88614200 -0.08157900

H 6.64093000 0.30838900 2.05181900

C 2.38164900 2.21583000 1.97843900

C 2.82542000 3.68653100 2.10736600

C 1.95241200 1.65729800 3.34971600

H 1.50767000 2.19357900 1.32210900

H 3.11118600 4.10609300 1.13675100

H 2.01279100 4.29986000 2.51548400

H 3.68641200 3.78853300 2.77879400

H 1.60664100 0.62116600 3.26454000

H 2.78148500 1.66918300 4.06680900

H 1.13854200 2.25467900 3.77559700

C 4.16716800 -0.93762600 -1.70957300

C 4.64756900 -2.39542100 -1.56253400

C 4.91294200 -0.26744300 -2.88254800

H 3.10465400 -0.96580100 -1.97090000

H 4.19851000 -2.88482300 -0.69197200

H 4.39036200 -2.97492900 -2.45690600

H 5.73576900 -2.45567700 -1.44828600

H 4.52938400 0.73433200 -3.09423500

H 5.98397600 -0.17784100 -2.66338500

H 4.80757600 -0.86655200 -3.79566100

C -2.55976000 1.69643100 -0.44810000

C -3.72358600 1.16596200 -1.06442500

C -2.69919300 2.51746100 0.70855900

C -4.98146200 1.46099000 -0.51849300

C -3.97894600 2.78429900 1.20772000

C -5.11984300 2.26210700 0.60636900

H -5.87134700 1.05934100 -0.99600800

H -4.08739500 3.41447600 2.08481200

H -6.10476000 2.48110300 1.01009400

C -3.68784300 0.30648700 -2.32533500

C -4.39066000 0.99479100 -3.51496100

C -4.32486300 -1.07739800 -2.08570100

H -2.63790400 0.15865900 -2.59997500

H -3.97888000 1.98862600 -3.71251300

H -4.28241200 0.39289100 -4.42571600

H -5.46348400 1.11425500 -3.32369600

H -3.83119900 -1.61736600 -1.27106100

H -5.38366700 -0.98540700 -1.81974400

H -4.27132100 -1.69614500 -2.98986500

C -1.48688800 3.14907700 1.38921000

C -1.63098500 3.26211200 2.91760400

C -1.16765500 4.53819900 0.79599200

H -0.62866000 2.50223700 1.18108000

H -1.90840900 2.30558200 3.37327900

H -0.68244700 3.58809200 3.35858500

H -2.38473500 4.00372000 3.20675300

H -0.94324200 4.48235700 -0.27269200

H -2.01634800 5.22055300 0.92900900

H -0.29764600 4.98058300 1.29712800

H 1.06189100 -3.73897400 1.15239600

B -0.05598000 -3.37756300 0.81318500

O -1.15628000 -4.13796600 1.34973000

O -0.34560900 -1.94398600 1.30977800

C -1.90782300 -3.38697500 2.30231900

C -1.01200900 -2.11201500 2.57882400

C -3.26776000 -3.02598900 1.67726600

H -3.73543500 -3.94243200 1.30297600

H -3.94951900 -2.56236600 2.39954500

H -3.15002000 -2.33788700 0.83400300

C -1.78677500 -0.83680600 2.91280800

H -2.38517400 -0.97105800 3.82136500

H -1.08770000 -0.01307500 3.09773000

H -2.45915900 -0.53721600 2.10412800

C 0.05631000 -2.35835900 3.65769400

H 0.76437400 -1.52277400 3.65791100

H -0.39100900 -2.42587300 4.65505800

H 0.61838800 -3.27487700 3.46396300

C -2.14902000 -4.26387400 3.53853600

H -2.66382200 -3.71046500 4.33376800

H -2.77689400 -5.11708400 3.26009700

H -1.21059800 -4.65853200 3.93497700

C 1.15372500 -3.33256700 -1.44352400

C -1.14020200 -2.43664000 -2.71591100

C 1.24071400 -2.90394400 -2.74698800

H 2.02975300 -3.60905900 -0.86310000

C 0.07055600 -2.27934200 -3.31870800

H -2.03676800 -1.97154500 -3.10755500

H 2.18727900 -2.91392900 -3.27164200

H 0.17854600 -1.64884800 -4.19988900

N -0.02792300 -3.30283200 -0.75008700

C -1.22531900 -3.45437500 -1.59840300

H -2.10816900 -3.32792700 -0.97173000

H -1.25990100 -4.48286500 -2.00536500

Ca 0.08484600 -0.63184900 -0.56384800

**18a-ts**

C -2.15468600 -2.66808900 -0.73187800

C -1.06002800 -3.55715800 -0.62405700

C 0.29015700 -3.37218400 -0.25369600

H -1.31831300 -4.58853100 -0.83556100

N -2.12302900 -1.35943900 -0.46880600

C -3.44126400 -3.32521200 -1.22357100

H -3.50366200 -4.36670200 -0.89661900

H -4.33605600 -2.79610900 -0.89149800

H -3.44916100 -3.32751800 -2.32082700

C 1.10117600 -4.65111500 -0.09221000

H 0.48372200 -5.53826800 -0.24683400

H 1.93756700 -4.68508200 -0.79792700

H 1.53914300 -4.70507800 0.91062500

N 0.87780600 -2.19183800 -0.04290600

C -3.33335400 -0.59442900 -0.44992100

C -3.99868700 -0.39799400 0.79639000

C -3.81612300 0.04550900 -1.62202600

C -5.12501800 0.43250400 0.83411300

C -4.94122800 0.87633800 -1.52119000

C -5.59506500 1.07314400 -0.31072100

H -5.64585300 0.58542100 1.77439700

H -5.31504700 1.36931500 -2.41482900

H -6.46964900 1.71628600 -0.25735600

C -3.52030600 -1.09906400 2.07129200

C -4.09633700 -2.52573900 2.20444100

C -3.83093600 -0.32013200 3.36311900

H -2.43037000 -1.20316100 1.99292900

H -3.77258400 -3.17789300 1.39116900

H -3.76707000 -2.98078500 3.14678400

H -5.19295400 -2.50011000 2.20745600

H -3.52943700 0.72974600 3.30073200

H -4.90005200 -0.34582400 3.60516500

H -3.30250700 -0.77712300 4.20787300

C -3.18868400 -0.17051600 -3.00005500

C -2.64465800 1.13393100 -3.61686200

C -4.18913800 -0.83508100 -3.97039800

H -2.34340900 -0.85702400 -2.88042000

H -1.83796200 1.56489000 -3.01433700

H -2.23648800 0.93539500 -4.61551500

H -3.43758400 1.88384100 -3.72640500

H -4.60613100 -1.75984700 -3.55838800

H -5.02811400 -0.16567100 -4.19495500

H -3.69541200 -1.07526100 -4.91959400

C 2.26090000 -2.14049100 0.32308800

C 3.26858500 -2.13582400 -0.67889700

C 2.61778700 -1.98643900 1.68932100

C 4.60364200 -1.97702500 -0.28846400

C 3.96846600 -1.83132700 2.02563000

C 4.96045600 -1.82693800 1.04918700

H 5.37981400 -1.96781600 -1.04899500

H 4.24769400 -1.71808200 3.07037000

H 6.00430900 -1.70958400 1.32834300

C 2.93876300 -2.28671200 -2.16425600

C 3.74965100 -3.41468200 -2.83350700

C 3.13739500 -0.96279100 -2.93069800

H 1.88116100 -2.55569800 -2.24598300

H 3.64064300 -4.36675600 -2.30183000

H 3.40978400 -3.56200500 -3.86541500

H 4.81936600 -3.17811200 -2.87278200

H 2.49980000 -0.16032200 -2.54315100

H 4.17462500 -0.61518600 -2.86147200

H 2.89434900 -1.09750300 -3.99211200

C 1.56799300 -1.99725300 2.79914700

C 1.53358400 -0.65940600 3.56489400

C 1.77016400 -3.17158300 3.77691300

H 0.59066500 -2.13417600 2.32475700

H 1.36844300 0.18695900 2.88752800

H 0.73148600 -0.66270400 4.31389100

H 2.47728600 -0.47386100 4.09137600

H 1.75675200 -4.13380600 3.25361100

H 2.72754800 -3.09399800 4.30579700

H 0.97342300 -3.18572000 4.53060300

H 0.33410100 1.54152100 -1.83036300

B 1.14840900 2.89512800 -0.14934800

O 0.44019000 4.07234600 -0.27641200

O 0.57047200 2.04896700 0.85633100

C -0.69753300 4.03943900 0.61010400

C -0.27016000 2.94905800 1.66844200

C -0.88022900 5.44087900 1.19744300

H -1.14940100 6.13630400 0.39639100

H -1.68541800 5.45375400 1.94132000

H 0.03738600 5.80598600 1.66424100

C 0.62696100 3.50187300 2.78277200

H 0.06462300 4.15261800 3.45996600

H 1.03382800 2.67027900 3.36638300

H 1.46593300 4.07109400 2.37185500

C -1.41743500 2.14544600 2.26507900

H -1.03091500 1.37582500 2.94211700

H -2.07313300 2.79920200 2.85090100

H -2.03921900 1.66736700 1.50197700

C -1.93631400 3.65867400 -0.21092900

H -2.84997900 3.67550900 0.39262000

H -2.04713000 4.38127800 -1.02505000

H -1.83494900 2.67360500 -0.67355700

C 3.39339400 1.82798500 -0.12745500

C 4.70976800 1.85076300 -0.44074500

H 2.99019800 1.10215700 0.57238400

C 4.38282400 3.82590900 -1.81049300

C 5.22899700 2.96854600 -1.21482300

H 5.37019300 1.09763600 -0.02837700

H 4.73656500 4.69933300 -2.35191400

H 6.30449600 3.12616200 -1.25723100

C 2.89784100 3.55446300 -1.79483400

H 2.32290400 4.48445600 -1.80205700

H 2.58900800 2.98497800 -2.69163900

N 2.50203200 2.78616800 -0.59302000

Ca -0.09058900 0.00001800 -0.39790800

**6b**

C 1.28849200 -0.00063500 1.81508300

C -0.00000500 -0.00078600 2.39611900

C -1.28850200 -0.00072400 1.81506100

H -0.00000500 -0.00193100 3.47961300

N 1.53486600 0.00095800 0.50341100

C 2.45597000 -0.00297400 2.79106200

H 3.09077400 -0.88266600 2.63666800

H 3.09630100 0.87229500 2.63466400

H 2.11349400 -0.00085900 3.82792100

C -2.45596300 -0.00313200 2.79106400

H -2.11347100 -0.00138400 3.82791700

H -3.09612000 0.87231300 2.63495100

H -3.09095000 -0.88264000 2.63642500

N -1.53488300 0.00088400 0.50342500

C 2.88090600 -0.00001400 0.01453700

C 3.52216500 1.22923400 -0.29215600

C 3.52035700 -1.23009400 -0.29257900

C 4.79053900 1.20083300 -0.88419300

C 4.78875500 -1.20333700 -0.88462300

C 5.42699600 -0.00166300 -1.17781400

H 5.28721600 2.13758800 -1.12398300

H 5.28404800 -2.14072900 -1.12478400

H 6.41119300 -0.00230900 -1.63840300

C 2.84911800 2.57398000 -0.02226400

C 2.44744000 3.26968900 -1.33984800

C 3.72009500 3.51114000 0.83611200

H 1.93145300 2.37872500 0.54174900

H 1.79902000 2.63629900 -1.96062700

H 1.91364400 4.20677500 -1.14004200

H 3.32850300 3.50646100 -1.94750800

H 4.00969400 3.03521200 1.77954400

H 4.63921700 3.80330100 0.31495000

H 3.17106100 4.42983600 1.07492800

C 2.84513400 -2.57388300 -0.02346600

C 3.71529800 -3.51379700 0.83272500

C 2.44064900 -3.26714200 -1.34145700

H 1.92842700 -2.37765000 0.54170600

H 4.00722200 -3.03949900 1.77626500

H 3.16463100 -4.43156800 1.07132300

H 4.63308900 -3.80742300 0.31004500

H 1.79255700 -2.63170600 -1.96046800

H 3.32053300 -3.50462800 -1.95054800

H 1.90536800 -4.20351000 -1.14228900

C -2.88090500 -0.00011200 0.01455200

C -3.52225800 1.22912500 -0.29194800

C -3.52025000 -1.23018100 -0.29277200

C -4.79061800 1.20072700 -0.88401400

C -4.78864200 -1.20344300 -0.88483300

C -5.42698200 -0.00177400 -1.17784500

H -5.28736600 2.13748200 -1.12366700

H -5.28385600 -2.14083600 -1.12515400

H -6.41117200 -0.00242300 -1.63845500

C -2.84931800 2.57388100 -0.02185500

C -3.72043500 3.51092200 0.83650800

C -2.44754900 3.26973300 -1.33933800

H -1.93169700 2.37864200 0.54222500

H -4.01015300 3.03488400 1.77984600

H -3.17145500 4.42960500 1.07550100

H -4.63949200 3.80312600 0.31525600

H -1.79900700 2.63645000 -1.96009600

H -3.32856500 3.50648100 -1.94707700

H -1.91385100 4.20684600 -1.13939800

C -2.84494300 -2.57396300 -0.02385300

C -2.44053800 -3.26708800 -1.34194000

C -3.71500300 -3.51399800 0.83231600

H -1.92820300 -2.37775400 0.54126600

H -1.79253300 -2.63157300 -1.96095900

H -1.90519400 -4.20344700 -1.14289700

H -3.32046200 -3.50457300 -1.95097500

H -4.00683900 -3.03982400 1.77594400

H -4.63284200 -3.80757200 0.30969100

H -3.16429800 -4.43179000 1.07074400

H 0.00003800 0.01313600 -3.45675500

Sr 0.00000700 0.00633900 -1.34314800

**13b**

C -1.29566700 -1.08174000 1.88660900

C 0.00000800 -1.20317400 2.44306900

C 1.29567000 -1.08194100 1.88655800

H 0.00005100 -1.58265900 3.45837200

N -1.57998700 -0.60636800 0.67270200

C -2.41816300 -1.60914400 2.77318800

H -3.30398500 -0.97073200 2.73475200

H -2.73333400 -2.60295000 2.43175800

H -2.09275300 -1.70142900 3.81196900

C 2.41822700 -1.60956800 2.77296600

H 2.09300700 -1.70161300 3.81183200

H 2.73295600 -2.60353900 2.43154500

H 3.30425600 -0.97144100 2.73421300

N 1.57997200 -0.60652600 0.67254200

C -2.92347700 -0.67798100 0.17850700

C -3.34378100 -1.82326300 -0.55012900

C -3.80601200 0.42570000 0.32421200

C -4.63641000 -1.84930900 -1.08699500

C -5.09170200 0.34466500 -0.22535200

C -5.51391400 -0.78196900 -0.92364500

H -4.96096900 -2.72420500 -1.64459200

H -5.77485500 1.18180300 -0.10426800

H -6.51564200 -0.82489700 -1.34303200

C -2.42386900 -3.02054400 -0.78520600

C -2.08463900 -3.18262500 -2.28168000

C -3.01399600 -4.32866400 -0.22216700

H -1.48638800 -2.83105900 -0.25275500

H -1.62363500 -2.27845200 -2.69637500

H -1.38699800 -4.01642400 -2.42853400

H -2.98449500 -3.39397900 -2.87201800

H -3.24603600 -4.24153000 0.84515100

H -3.93882200 -4.60853500 -0.74037200

H -2.30250300 -5.15371400 -0.34765800

C -3.39879600 1.70159900 1.05909900

C -4.21217800 1.91945400 2.35141800

C -3.51573600 2.94109400 0.14925200

H -2.34768200 1.59284700 1.34781200

H -4.10317400 1.08068000 3.04654300

H -3.87904900 2.82863100 2.86742600

H -5.28062300 2.03369600 2.13288100

H -2.94103700 2.81761000 -0.77489600

H -4.55716200 3.13343500 -0.13309100

H -3.14441000 3.83532600 0.66486300

C 2.92348500 -0.67810300 0.17825200

C 3.34358800 -1.82315200 -0.55092800

C 3.80621400 0.42541600 0.32445500

C 4.63616300 -1.84908000 -1.08793000

C 5.09185200 0.34441100 -0.22525900

C 5.51380100 -0.78194400 -0.92414500

H 4.96053300 -2.72374700 -1.64598100

H 5.77514000 1.18138200 -0.10380100

H 6.51546900 -0.82479600 -1.34365400

C 2.42350800 -3.02024600 -0.78633000

C 3.01327700 -4.32846100 -0.22312800

C 2.08458400 -3.18221900 -2.28287000

H 1.48593600 -2.83057600 -0.25406700

H 3.24504300 -4.24131900 0.84425500

H 2.30168500 -5.15340200 -0.34879000

H 3.93819900 -4.60846500 -0.74108100

H 1.62383000 -2.27794200 -2.69763300

H 2.98452400 -3.39372300 -2.87303000

H 1.38682000 -4.01588100 -2.42989600

C 3.39919700 1.70105900 1.05993600

C 3.51602900 2.94087600 0.15052400

C 4.21278400 1.91835300 2.35221100

H 2.34810400 1.59220600 1.34876900

H 2.94109500 2.81775400 -0.77353300

H 3.14488500 3.83495000 0.66654700

H 4.55740900 3.13322300 -0.13198100

H 4.10389600 1.07924900 3.04697300

H 5.28119800 2.03270900 2.13356200

H 3.87971500 2.82728700 2.86868700

H -0.00018000 0.17431300 -3.10282400

C 0.00044600 3.70561300 0.06084000

C 0.00033900 5.08696200 -0.11082000

C -0.00015000 5.59839400 -1.40965300

C -0.00049800 4.70869300 -2.48293000

C -0.00034100 3.33952000 -2.22003900

N 0.00011200 2.83988000 -0.96839000

H -0.00024800 6.67144100 -1.57996700

H 0.00084100 3.26880700 1.05636100

H 0.00064400 5.74184500 0.75460300

H -0.00087700 5.06059000 -3.50952700

H -0.00057400 2.59676700 -3.01751700

Sr 0.00002300 0.26197300 -0.96086500

**14b-ts**

C -1.07470300 -2.68295000 0.07997700

C 0.24346300 -3.18035600 0.16860800

C 1.48479500 -2.50460600 0.18786000

H 0.31499900 -4.25865000 0.24976100

N -1.38859500 -1.39244100 -0.02208500

C -2.18638200 -3.72143400 0.11913900

H -2.82183600 -3.58022600 1.00088100

H -2.83976600 -3.62568900 -0.75512200

H -1.78735600 -4.73785100 0.14394100

C 2.71647000 -3.38533400 0.34074900

H 3.34046000 -3.34295300 -0.55899200

H 3.34278000 -3.04178300 1.17085100

H 2.44515000 -4.42814300 0.51879100

N 1.63117700 -1.18498800 0.08085500

C -2.73999300 -0.94104700 -0.03672800

C -3.37463400 -0.64124200 -1.27415300

C -3.39900100 -0.63557900 1.18797800

C -4.63766800 -0.03514900 -1.25821700

C -4.65998800 -0.02942000 1.14564600

C -5.28042000 0.27461900 -0.06359100

H -5.12720200 0.19531800 -2.20116300

H -5.16718200 0.20897000 2.07731100

H -6.25916100 0.74668700 -0.07392100

C -2.72964100 -0.98762200 -2.61593100

C -2.45472900 0.26432400 -3.47376600

C -3.57958400 -2.00196600 -3.40831600

H -1.76724200 -1.46622900 -2.40604600

H -1.79558700 0.98194200 -2.96771600

H -1.97302100 -0.01703600 -4.41805200

H -3.38329500 0.79344200 -3.71847700

H -3.77278300 -2.90823500 -2.82374900

H -4.54975900 -1.57722700 -3.69236900

H -3.06320500 -2.29635500 -4.32980100

C -2.76268800 -0.94881500 2.54225500

C -3.68076800 -1.80566600 3.43615300

C -2.34475600 0.33561800 3.28651300

H -1.85316200 -1.52798000 2.35823400

H -3.99679400 -2.72122100 2.92431200

H -3.15617300 -2.09455400 4.35466500

H -4.58580200 -1.26179700 3.73127200

H -1.62057100 0.92564900 2.70956500

H -3.20850300 0.98218800 3.48199400

H -1.87902900 0.09428600 4.24963500

C 2.91932100 -0.56974300 0.11048000

C 3.64572900 -0.37011200 -1.09680000

C 3.40853400 -0.02079100 1.32885500

C 4.83491600 0.36769700 -1.05547600

C 4.60535500 0.70696900 1.31278000

C 5.31813900 0.90555800 0.13430100

H 5.39350700 0.52523900 -1.97476600

H 4.98557700 1.12453800 2.24183200

H 6.24366600 1.47510400 0.14242700

C 3.16542000 -0.93087900 -2.43500100

C 4.21026000 -1.86176900 -3.08290200

C 2.77280800 0.19248700 -3.41671200

H 2.26869900 -1.52845800 -2.24352800

H 4.50463000 -2.67415400 -2.40921700

H 3.80468200 -2.31064200 -3.99750900

H 5.12007300 -1.31594400 -3.35938100

H 1.99670800 0.84905000 -3.00306700

H 3.63568600 0.82274400 -3.66424800

H 2.39202500 -0.23309400 -4.35343400

C 2.67658800 -0.21788200 2.65638100

C 2.25555000 1.12278100 3.29333900

C 3.51617900 -1.04170100 3.65399800

H 1.76675800 -0.79157900 2.45040700

H 1.63727100 1.72929300 2.61973200

H 1.68303700 0.94606900 4.21228800

H 3.12932500 1.72866600 3.56074200

H 3.79710700 -2.01475200 3.23656600

H 4.43990300 -0.51762000 3.92663400

H 2.95011800 -1.22020300 4.57628600

H 0.31916700 2.38578100 -1.72491100

C -1.24697600 3.61673300 0.89405600

C -1.82416800 4.67601000 0.21872500

C -1.05004100 5.32245300 -0.78021100

C 0.17269000 4.81048400 -1.12122400

C 0.60620800 3.57662600 -0.52306900

N -0.02286300 3.10400100 0.61899300

H -1.41782600 6.23207100 -1.24939100

H -1.75774300 3.17437500 1.74933900

H -2.79697700 5.05315500 0.51577500

H 0.79677000 5.27613600 -1.87808200

H 1.66761100 3.34736100 -0.57674900

Sr -0.00240700 0.69101400 -0.19216000

**15b**

C 1.25395600 -0.27450000 2.48118600

C -0.03290300 -0.33273500 3.05903600

C -1.31092000 -0.26289100 2.46248300

H -0.04144000 -0.46816400 4.13415600

N 1.49059900 -0.10232300 1.18109100

C 2.42383800 -0.42771600 3.44211900

H 3.01279700 -1.31955400 3.19982400

H 3.10714000 0.42545200 3.36891900

H 2.08510900 -0.51193900 4.47697700

C -2.49606700 -0.39569200 3.40772600

H -2.17347700 -0.47010400 4.44848300

H -3.17027900 0.46273400 3.31349900

H -3.08927400 -1.28513900 3.16776700

N -1.52692300 -0.09491300 1.15841000

C 2.82013300 -0.07529800 0.66205000

C 3.47274600 1.17218200 0.45787500

C 3.43211400 -1.27630900 0.20760700

C 4.70925600 1.19202300 -0.19930000

C 4.66770800 -1.19898100 -0.44668600

C 5.30681400 0.02037600 -0.65512200

H 5.21383200 2.14284500 -0.35426000

H 5.13922400 -2.11301000 -0.79858700

H 6.26557300 0.05646900 -1.16545700

C 2.86611400 2.48715600 0.94603000

C 2.54629400 3.44464100 -0.22047800

C 3.76981200 3.19318800 1.97674700

H 1.92333900 2.24982100 1.44961900

H 1.88996200 2.97981400 -0.96828000

H 2.05229300 4.35218300 0.14748100

H 3.45714400 3.75146800 -0.74796900

H 3.98683500 2.54367400 2.83161800

H 4.72774200 3.49003500 1.53384300

H 3.28357100 4.10016400 2.35581600

C 2.78164100 -2.64382500 0.41716700

C 3.69811800 -3.60344800 1.20299500

C 2.35335700 -3.28889200 -0.91710100

H 1.87779900 -2.49409000 1.01547800

H 4.01870300 -3.16764800 2.15587600

H 3.17092000 -4.54021900 1.41933700

H 4.60072800 -3.85733500 0.63468700

H 1.63165700 -2.67332600 -1.46757100

H 3.21655100 -3.44982800 -1.57403900

H 1.88503000 -4.26430600 -0.73754100

C -2.84804900 -0.06147900 0.61848200

C -3.48605400 1.19021200 0.39604800

C -3.46215100 -1.26033700 0.15993200

C -4.71219800 1.21738000 -0.27953100

C -4.68774100 -1.17529400 -0.51253400

C -5.31337200 0.04821900 -0.73675100

H -5.20465000 2.17179300 -0.45015000

H -5.16162800 -2.08716500 -0.86681100

H -6.26395400 0.08958600 -1.26171500

C -2.86739200 2.50249600 0.87583200

C -3.78210500 3.24685500 1.86895700

C -2.49696100 3.42858700 -0.30123200

H -1.94290600 2.25683800 1.40845500

H -4.03289000 2.61864200 2.73060200

H -3.28741500 4.15161000 2.24232300

H -4.72272800 3.55350900 1.39665600

H -1.83889000 2.93250700 -1.02797000

H -3.38798200 3.74815900 -0.85434200

H -1.98721500 4.33009000 0.06020200

C -2.83034600 -2.63426400 0.38589100

C -2.42568400 -3.31280500 -0.93947200

C -3.75729500 -3.56384700 1.19606200

H -1.91918100 -2.48890000 0.97447600

H -1.69882500 -2.72360700 -1.51118400

H -1.97472100 -4.29305400 -0.74189400

H -3.29804500 -3.47400900 -1.58418300

H -4.06034600 -3.10819000 2.14527300

H -4.67020400 -3.80701600 0.63955700

H -3.24676200 -4.50773000 1.42136000

C -1.16414600 -0.25432500 -3.29207600

C -1.07547300 1.12365400 -3.55252500

H -2.13998100 -0.67785200 -3.03025500

C 1.33862600 0.90188600 -3.41612100

C 0.23250700 1.71125900 -3.45456000

H -1.96570800 1.72965900 -3.68143800

H 2.34008600 1.29268900 -3.24973200

H 0.32655100 2.79101400 -3.33113100

N -0.10985600 -1.06529200 -3.10897400

C 1.11058800 -0.55031900 -3.77945600

H 0.98968400 -0.66068500 -4.87673100

H 1.95955200 -1.17937700 -3.49075000

Sr -0.00180700 0.22411100 -0.80636000

**16b-ts**

C -3.03765600 -1.87089000 0.75193600

C -2.38614100 -3.11724300 0.81536900

C -1.03549200 -3.42293900 0.57391100

H -3.01508100 -3.95830600 1.07688700

N -2.43510800 -0.71104900 0.45620700

C -4.53900600 -1.88228400 1.04583100

H -4.77394600 -1.17518300 1.84811100

H -5.10413100 -1.56791100 0.16190200

H -4.87448400 -2.87832200 1.34037500

C -0.63913200 -4.89304200 0.72076200

H -0.17497400 -5.24977100 -0.20320300

H 0.09682100 -5.01636400 1.52201800

H -1.50850400 -5.51396400 0.94405400

N -0.10726100 -2.51866300 0.23279300

C -3.17398700 0.51776700 0.29733300

C -3.15924000 1.48702400 1.33546700

C -3.75720800 0.83580000 -0.95931700

C -3.69348000 2.75727300 1.08734800

C -4.27115600 2.12120000 -1.16280500

C -4.23441900 3.08197600 -0.15414400

H -3.68442100 3.50190900 1.87536800

H -4.70482400 2.37292400 -2.12462800

H -4.63211400 4.07454800 -0.33256000

C -2.62443500 1.14667500 2.72949700

C -3.79074000 1.05431800 3.74654300

C -1.56798300 2.16627600 3.22362700

H -2.15314900 0.16014000 2.66727000

H -4.52777500 0.31338700 3.42182800

H -3.41239700 0.76137500 4.73317300

H -4.29276200 2.02338200 3.84040400

H -0.70787500 2.20681900 2.54949600

H -1.99992800 3.17066600 3.29354700

H -1.21184500 1.87899100 4.22053300

C -3.83240700 -0.19577900 -2.08895400

C -2.93824100 0.21662600 -3.28472300

C -5.29301700 -0.40598700 -2.56379900

H -3.45959000 -1.14687600 -1.70232900

H -1.88975000 0.30546300 -2.98260000

H -3.00372600 -0.53099100 -4.08418600

H -3.25585100 1.18504200 -3.68585400

H -5.93674800 -0.70014300 -1.72775800

H -5.69671900 0.51233700 -3.00310000

H -5.32810300 -1.19351100 -3.32540500

C 1.28732800 -2.83603200 0.08330200

C 1.85467000 -2.94869900 -1.21659200

C 2.13696800 -2.83496600 1.22658100

C 3.24880200 -2.99994900 -1.34598100

C 3.52306200 -2.88744300 1.04526500

C 4.08189800 -2.95475400 -0.23051100

H 3.68502900 -3.08325900 -2.33469400

H 4.17362300 -2.88283800 1.91339500

H 5.15859900 -2.98947500 -0.35285800

C 0.96779500 -3.10699400 -2.45691200

C 0.95200500 -4.59743100 -2.89507600

C 1.40961000 -2.22896500 -3.65268900

H -0.05120000 -2.82530600 -2.17219800

H 0.60724800 -5.24397400 -2.08320300

H 0.28505700 -4.73110800 -3.75489700

H 1.96133400 -4.91227800 -3.18332400

H 1.45187600 -1.16950800 -3.38752900

H 2.39762900 -2.53267800 -4.01680000

H 0.69796800 -2.35301500 -4.47727500

C 1.55842100 -2.80577600 2.64574700

C 1.88188300 -1.47791700 3.37294100

C 2.07044900 -4.00142700 3.48882100

H 0.47231100 -2.88308900 2.56837900

H 1.45990700 -0.62189700 2.83433300

H 1.45736800 -1.48505100 4.38390500

H 2.96536200 -1.33453000 3.45118600

H 1.85038900 -4.95114000 2.99065600

H 3.15298000 -3.93741600 3.64366300

H 1.58425800 -4.00149200 4.47134100

H -0.50183200 2.83081500 -0.09684500

B 0.65909600 2.82619300 0.16011300

O 1.44690200 3.95964500 0.11323400

O 1.23074500 1.80952400 1.01836400

C 2.76484700 3.71158300 0.77917900

C 2.57812800 2.30319400 1.51886100

C 3.82195900 3.71405700 -0.33273000

H 3.78454200 4.68917000 -0.82693200

H 4.82447200 3.56579000 0.08141700

H 3.60852000 2.95403500 -1.08773200

C 3.63751700 1.26367000 1.13911000

H 4.62035900 1.58443000 1.50121700

H 3.39601400 0.30337400 1.60299900

H 3.68507000 1.12303000 0.05908400

C 2.44825000 2.40625200 3.04354300

H 2.23222900 1.41406700 3.44792600

H 3.38210800 2.76790100 3.48427100

H 1.63510000 3.07703600 3.32702100

C 2.98277200 4.89975800 1.72512800

H 3.93347600 4.80419900 2.25935400

H 3.00536100 5.81184500 1.12267100

H 2.16776000 4.98917400 2.44591200

C 2.10560700 0.99643900 -2.20922100

C 2.91083100 1.64542700 -3.11216400

H 2.52970500 0.17561600 -1.62343500

C 1.14573500 3.27187800 -3.47488700

C 2.43550400 2.91461400 -3.63389500

H 3.92340700 1.30848400 -3.29725400

H 0.77703500 4.25751700 -3.73835300

H 3.15681200 3.60500600 -4.06322400

N 0.80288500 1.34552800 -1.89750200

C 0.17105300 2.23045000 -2.94715200

H -0.71064500 2.70419200 -2.50176700

H -0.17827400 1.59667700 -3.78520200

Sr -0.10203900 -0.04993200 -0.06762300

**17b**

C -1.88782000 -1.78550000 -1.54786500

C -0.66846900 -2.10670500 -2.22947400

C 0.69746600 -2.17812700 -1.84085600

H -0.85743800 -2.68765800 -3.12743800

N -2.02042000 -0.90627000 -0.56307600

C -3.07039900 -2.61757300 -2.03838500

H -2.95068800 -3.65188900 -1.69274700

H -4.03006500 -2.25065100 -1.67641100

H -3.08813800 -2.64787400 -3.13275000

C 1.52837200 -3.09748300 -2.73903000

H 1.71516100 -2.60159300 -3.70011000

H 2.49318800 -3.34464400 -2.29558900

H 0.99174700 -4.02612100 -2.95352700

N 1.26220600 -1.52645500 -0.82014900

C -3.27400300 -0.77794700 0.12713400

C -3.47586500 -1.48984900 1.33698800

C -4.29213800 0.08794000 -0.35620000

C -4.70207600 -1.37153000 2.00215800

C -5.50259900 0.16395700 0.34551000

C -5.72004100 -0.56246400 1.51083000

H -4.86061100 -1.92920800 2.92142000

H -6.29142100 0.80914200 -0.03137800

H -6.66882300 -0.48952700 2.03552200

C -2.40179500 -2.38937600 1.93889800

C -2.81095500 -3.87495900 1.88537200

C -2.05467600 -1.96740800 3.38004600

H -1.49806800 -2.27398400 1.33429100

H -3.02040500 -4.19420400 0.85833300

H -2.01182600 -4.51274100 2.28255300

H -3.71271200 -4.06185000 2.48095100

H -1.74551000 -0.91660700 3.42105100

H -2.90982200 -2.08616000 4.05553900

H -1.23571500 -2.57858600 3.77440400

C -4.13193700 0.94862100 -1.60863900

C -4.47119700 2.42685200 -1.32601600

C -4.98357100 0.44176000 -2.79126600

H -3.08214000 0.90268500 -1.91852600

H -3.94302300 2.80162800 -0.44310700

H -4.20244600 3.05332000 -2.18515000

H -5.54372100 2.56664400 -1.14928200

H -4.71929800 -0.57926000 -3.08063300

H -6.05023300 0.45130400 -2.53679400

H -4.84468500 1.08709900 -3.66754700

C 2.54992900 -1.92987500 -0.33356700

C 3.74791900 -1.31486700 -0.78341200

C 2.61910000 -2.94182400 0.66337300

C 4.97284900 -1.73129900 -0.24359500

C 3.86878500 -3.32536800 1.16279500

C 5.04491200 -2.73034700 0.71904300

H 5.89082900 -1.26921400 -0.59713600

H 3.92185700 -4.11004900 1.91227200

H 6.00615600 -3.04279700 1.11808500

C 3.77671100 -0.24026500 -1.86691000

C 4.45373900 -0.74430300 -3.15863900

C 4.46943300 1.04654700 -1.37718800

H 2.74056800 0.00964700 -2.12298100

H 3.96847600 -1.64649100 -3.54339500

H 4.41452000 0.02415900 -3.94070900

H 5.50859300 -0.98461900 -2.98156200

H 3.97912000 1.45659500 -0.48767900

H 5.51691700 0.86075700 -1.11595100

H 4.46325800 1.81668100 -2.15743800

C 1.37104200 -3.65040500 1.18189400

C 1.33111300 -3.71755900 2.72000500

C 1.23682400 -5.06440200 0.58010600

H 0.50467700 -3.06917600 0.85467200

H 1.41341200 -2.71994600 3.16569900

H 0.38823200 -4.16510000 3.05465900

H 2.14143400 -4.33287400 3.12765900

H 1.20777700 -5.03480900 -0.51412800

H 2.08242700 -5.69873700 0.87340800

H 0.31647400 -5.54805300 0.93000100

H -1.17944700 3.70552100 1.00780000

B 0.00205200 3.48475800 0.76869800

O 0.94741100 4.34143700 1.43687800

O 0.40198800 2.07590200 1.24936900

C 1.68008700 3.64553300 2.44397800

C 0.93194700 2.25470500 2.57818900

C 3.13553800 3.49474400 1.96529000

H 3.51703500 4.48396600 1.69239200

H 3.78912200 3.07590100 2.73930600

H 3.20087800 2.85456500 1.08009600

C 1.83134900 1.06518500 2.92171800

H 2.31489400 1.20719200 3.89516600

H 1.23297600 0.14779000 2.98232900

H 2.61306300 0.91020400 2.17299200

C -0.24870300 2.30255800 3.56357700

H -0.82980700 1.37829500 3.46926900

H 0.09481200 2.38428800 4.60024700

H -0.91416700 3.14185400 3.34782200

C 1.67014600 4.48661400 3.72816400

H 2.16816100 3.96753300 4.55668100

H 2.20417300 5.42573900 3.54854100

H 0.65119500 4.73783500 4.03188800

C -0.99614600 3.35074700 -1.57039400

C 1.46621400 2.67293800 -2.65105300

C -0.94657800 2.86145800 -2.85795300

H -1.93814300 3.54799800 -1.06707800

C 0.32978100 2.37987000 -3.33813600

H 2.43296000 2.29343400 -2.96524500

H -1.84882900 2.76346700 -3.44962700

H 0.36164200 1.73945100 -4.21847900

N 0.11693000 3.47581200 -0.79242100

C 1.37896000 3.68312900 -1.52668400

H 2.20357800 3.59211500 -0.81875900

H 1.40924000 4.71574400 -1.92313500

Sr -0.02414000 0.63830100 -0.66719800

**18b-ts**

C -2.06325800 -2.88811100 -0.81214700

C -0.94794000 -3.75187000 -0.75749400

C 0.39275700 -3.51486000 -0.38203200

H -1.17650400 -4.78898300 -0.97518400

N -2.02432300 -1.57626500 -0.58290000

C -3.39365100 -3.56919500 -1.10851300

H -3.92560200 -3.79702200 -0.17626300

H -4.05085800 -2.93098500 -1.70405700

H -3.24220500 -4.51208100 -1.63988600

C 1.27209000 -4.75379400 -0.27202900

H 0.69454200 -5.66874300 -0.42009500

H 2.07799800 -4.73368000 -1.01352000

H 1.75382700 -4.80179400 0.71082300

N 0.90983500 -2.31561200 -0.11440000

C -3.21415800 -0.79509600 -0.49632100

C -3.90629200 -0.69993900 0.74566800

C -3.63203100 -0.00140300 -1.60193600

C -4.94060600 0.23513500 0.87490500

C -4.67949700 0.91159600 -1.41739100

C -5.32097800 1.04891900 -0.18987800

H -5.46672900 0.31968900 1.82295600

H -5.00383900 1.52001100 -2.25754900

H -6.12719500 1.76787700 -0.06957500

C -3.58012600 -1.61928500 1.92468700

C -4.82289800 -2.40234500 2.39549700

C -2.95119400 -0.86520500 3.11151400

H -2.84263100 -2.35117700 1.58500200

H -5.29359000 -2.94166300 1.56659100

H -4.54413900 -3.13348500 3.16386200

H -5.58042300 -1.73966700 2.83036100

H -1.99926800 -0.39804100 2.83310800

H -3.61248700 -0.07291900 3.48231900

H -2.75143200 -1.55224800 3.94286700

C -3.03636700 -0.17701100 -3.00035700

C -2.49965400 1.13378600 -3.60677100

C -4.07065300 -0.81630700 -3.95281700

H -2.19475800 -0.87274800 -2.91724300

H -1.68049600 1.55056700 -3.01061400

H -2.10923300 0.94493400 -4.61430800

H -3.28896100 1.89017900 -3.69785800

H -4.44954400 -1.76918000 -3.56766700

H -4.93135200 -0.15329400 -4.10288100

H -3.61858900 -1.00431300 -4.93397700

C 2.27007000 -2.19187900 0.29914900

C 3.31725800 -2.11335900 -0.66017600

C 2.56295000 -2.02851400 1.68138000

C 4.62511800 -1.89834700 -0.21159900

C 3.88997400 -1.81404300 2.07581500

C 4.91997800 -1.75506300 1.14213600

H 5.43040800 -1.83491000 -0.93826600

H 4.12032600 -1.70143400 3.13259000

H 5.94529700 -1.59553800 1.46568000

C 3.05007600 -2.23332100 -2.16034200

C 3.95708100 -3.27774400 -2.84102900

C 3.18541800 -0.86829100 -2.86674500

H 2.01562600 -2.56518000 -2.28999400

H 3.89358900 -4.25576700 -2.35032600

H 3.66337600 -3.40524900 -3.88957200

H 5.00951500 -2.97080600 -2.83238000

H 2.47890600 -0.12793300 -2.47378000

H 4.19358700 -0.45556400 -2.74356000

H 2.99052100 -0.97442100 -3.94121500

C 1.47180100 -2.10797600 2.74857800

C 1.34940700 -0.79563900 3.55020900

C 1.68448100 -3.29966200 3.70333600

H 0.51994500 -2.27626000 2.23357900

H 1.17362700 0.07223900 2.89965100

H 0.51995700 -0.85437000 4.26565600

H 2.26320000 -0.58425600 4.11784900

H 1.73328300 -4.24491800 3.15228900

H 2.61638800 -3.19721400 4.27192700

H 0.86000700 -3.36956500 4.42338400

H 0.33369900 1.58742700 -1.72813700

B 1.05198100 3.07256700 -0.07147300

O 0.39343200 4.24840200 -0.36418900

O 0.39057000 2.34385100 0.96315500

C -0.81794400 4.31164300 0.41682500

C -0.50626400 3.32007800 1.60555500

C -1.02594900 5.76429600 0.85063500

H -1.20076300 6.38337000 -0.03485500

H -1.89911500 5.85916400 1.50687600

H -0.15031300 6.16140500 1.36909700

C 0.30061600 3.96324900 2.74083800

H -0.30490800 4.67595900 3.31005600

H 0.64199800 3.18060100 3.42627300

H 1.18148400 4.48569400 2.35596300

C -1.72049300 2.59523400 2.17311600

H -1.41364600 1.90919000 2.96979900

H -2.41904200 3.31550500 2.61381400

H -2.27153700 2.03156700 1.41473600

C -1.98460500 3.86292500 -0.47164700

H -2.94515300 3.93332000 0.04967800

H -2.02080600 4.51269900 -1.35144700

H -1.84724200 2.84109900 -0.83401900

C 3.26511600 1.98850900 0.24156500

C 4.59273600 1.91578700 -0.01216900

H 2.80972700 1.41847700 1.04636300

C 4.38930900 3.63279800 -1.71495900

C 5.18008800 2.86061100 -0.95049400

H 5.21010600 1.22970300 0.55445300

H 4.79823900 4.38421600 -2.38535200

H 6.26211500 2.96848400 -0.98168000

C 2.89385800 3.43062200 -1.71107100

H 2.36540900 4.37341700 -1.87944000

H 2.57901600 2.74675100 -2.52144800

N 2.42440400 2.87095000 -0.42272400

Sr -0.08443200 0.00515600 -0.19306800
